# Structural basis for lipid-mediated activation of G protein-coupled receptor GPR55

**DOI:** 10.1038/s41467-025-57204-y

**Published:** 2025-02-25

**Authors:** Tobias Claff, Rebecca Ebenhoch, Jörg T. Kley, Aniket Magarkar, Herbert Nar, Dietmar Weichert

**Affiliations:** Boehringer Ingelheim Pharma GmbH & Co. KG, Global Medicinal Chemistry, Biberach an der Riß, Germany

**Keywords:** Cryoelectron microscopy, Membrane lipids, Permeation and transport

## Abstract

GPR55 is an orphan G protein-coupled receptor (GPCR) and represents a promising drug target for cancer, inflammation, and metabolic diseases. The endogenous activation of lipid GPCRs can be solely mediated by membrane components and different lipids have been proposed as endogenous activators of GPR55, such as cannabinoids and lysophosphatidylinositols. Here, we determine high-resolution cryo-electron microscopy structures of the activated GPR55 in complex with heterotrimeric G_13_ and two structurally diverse ligands: the putative endogenous agonist 1-palmitoyl-2-lysophosphatidylinositol (LPI) and the synthetic agonist ML184. These results reveal insights into ligand recognition at GPR55, G protein coupling and receptor activation. Notably, an orthosteric binding site opening towards the membrane is observed in both structures, enabling direct interaction of the agonists with membrane lipids. The structural observations are supported by mutagenesis and functional experiments employing G protein dissociation assays. These findings will be of importance for the structure-based development of drugs targeting GPR55.

## Abstract (web summary)

Endogenous activation of lipid GPCRs can be mediated by membrane components. Here, authors show an orthosteric binding site opens towards the membrane, setting the foundation for structure-based development of drugs targeting GPR55.

## Introduction

G protein-coupled receptors (GPCRs) constitute the largest family of human membrane proteins and represent the most prominent and clinically relevant drug target class^1,2^. The composition and fluidity of cellular membranes play a crucial role in influencing the dynamic nature of GPCR-lipid interactions^3^, thereby affecting signal transduction pathways in (patho)physiological conditions. Several GPCRs have been identified to be activated by a diverse array of endogenous lipids, such as the sphingosine-1-phosphate receptors (S1PR), the cannabinoid receptors, the lysophosphatidic acid receptors (LPAR), the leukotriene receptors, and the prostaglandin receptors^4^. However, despite tremendous efforts to discover new endogenous GPCR ligands^5,6^, ~35% of all non-sensory GPCRs currently remain orphan, i.e., without known physiological agonist^7^.

GPR55 belongs to one of currently 87 class A orphan GPCRs^7^ and has attracted significant interest ever since it was reported to respond to endocannabinoid lipids^8^. In contrast to many GPCRs that signal via multiple different G proteins^9^, GPR55 has been reported to selectively couple to Gα_13_ proteins^10,11^. Mediated by small guanosine triphosphate hydrolases (small GTPases), Gα_13_ proteins initiate a signaling cascade which activates Rho kinases (ROCK) to phosphorylate various substrates responsible for the modulation of inflammation, cytoskeletal remodeling, and migration^12^. GPR55 is highly expressed in adrenal tissue and several brain areas but also in the gastrointestinal tract, liver, and immune cells^11,13,14^. Consequently, GPR55 has been implicated as a potential target for the treatment of Parkinson’s disease^15^, metabolic disorders^16,17^, neuropathic pain^18,19^, cancer^20^, and inflammatory diseases^21^.

**Fig. 1:**
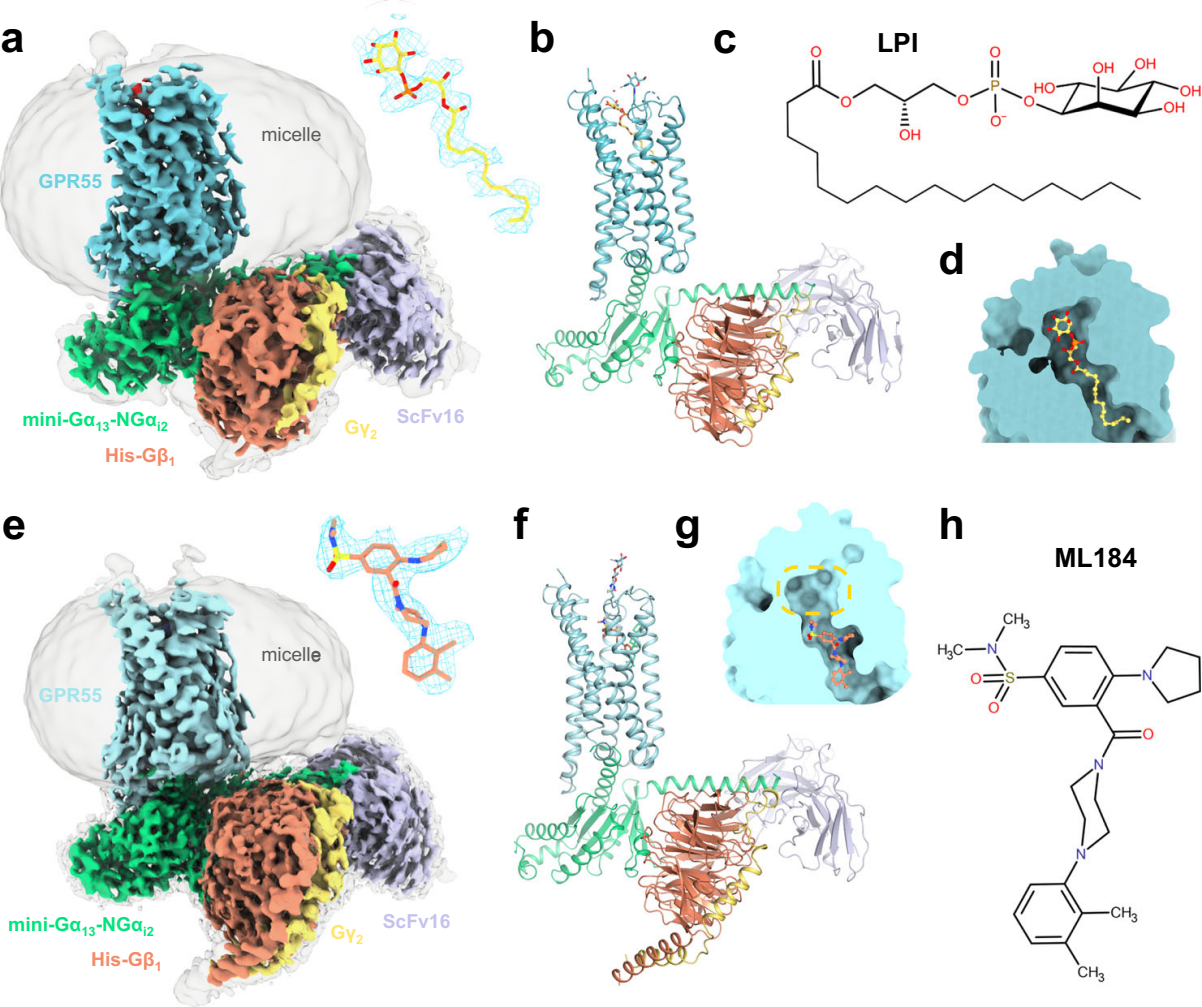
Architecture of the GPR55-G_13_ signaling complex with lipid and synthetic agonists. **a** Cryo-EM map (consensus, EMDB-51285) of the GPR55-Gα_13_β_1_γ_2_-ScFv16-LPI complex at two different contour levels. The enlarged cryo-EM map for LPI (yellow sticks) is shown in blue mesh. **b** Full model corresponding to the signaling complex of (**a**) (shown as cartoon representation). **c** Chemical structure of LPI. **d** Overview of ligand binding pocket position of LPI. **e** Cryo-EM map (consensus, EMDB-51281) of the GPR55-Gα_13_β_1_γ_2_-ScFv16-ML184 complex at two different contour levels. The enlarged cryo-EM map for ML184 (salmon sicks) is shown in blue mesh. **f** Full model corresponding to the signaling complex of (**d**). **g** Overview of ligand binding pocket position of ML184. The yellow circle highlights the position of the polar head group of LPI. **h** Chemical structure of ML184.

GPR55 was suggested to be a novel, atypical cannabinoid receptor that possibly explained cannabinoid effects unrelated to the established cannabinoid receptors, such as vasodilation^11,22^. However, the classification remains controversial due to inconsistency and limited reproducibility of the experimental findings^23–25^. The discovery of hydrolysis products of the membrane lipid phosphatidylinositol as agonists of GPR55 provided an alternative^26–28^. It was found that GPR55 can recognize lysophosphatidylinositols with varying fatty acid substitution, such as 1-palmitoyl-2-lysophosphatidylinositol (referred to as LPI, Fig. 1c)^26^, 2-arachidonoyl-1-lysophosphatidylinositol (2-AG-PI)^27^, and 1-arachidonoyl-2-lysophosphatidylinositol (1-AG-PI)^10^. In this study, we used a natural product mixture of different lysophosphatidylinositols produced from soybeans by hydrolysis with phospholipase A_2_. Although the abbreviation LPI is generally used for the lysophosphatidylinositol mixture containing various fatty acid substituents^29^, herein, the main product of the hydrolysis reaction^30^, 1-palmitoyl-2-lysophosphatidylinositol, is referred to as LPI throughout the manuscript for simplicity. The lysophosphatidylinositol findings corroborate that GPR55 can be activated by membrane components in vitro. However, the receptor currently retains its orphan status due to the lack of significant in-vivo evidence for any of the discovered lipids^31^. Simultaneous to the efforts in revealing the endogenous GPR55 ligand, screening campaigns and medicinal chemistry design programs have discovered synthetic GPR55 agonists with sub-micromolar potency^32–36^, such as the sulfonamide 3-[[4-(2,3-dimethylphenyl)-1-piperazinyl]carbonyl]-*N*,*N*-dimethyl-4-(1-pyrrolidinyl)benzenesulfonamide (ML184) (Fig. 1h) with a reported potency of 260 nM as determined in β-arrestin recruitment assays^32^.

Technological advances in cryo-electron microscopy (cryo-EM) have revolutionized the determination of GPCR structures^37^, accelerating drug design and the understanding of ligand pharmacology^38,39^. Thus far, experimental structures of GPR55 have not been reported, and the binding modes of ligands with different scaffolds remain elusive. In addition, few high-resolution structures of GPCRs in complex with G proteins from the Gα_12/13_-family have been published. As of July 2024, nine structures are available (Supplementary Table S1), of which the majority has been determined with class B adhesion receptors^40^. Recently, structures of class A GPCRs in complex with G_13_ have been obtained for GPR35^41^ and for the S1PR_2_^42^, both at moderate overall resolution of 3.2 Å. GPR35 and S1PR_2_ are related to GPR55 (49% and 33% sequence similarity, respectively). In contrast to GPR55, it has been shown that both receptors also couple to Gα_12_ and to the Gα_i_-family^10^.

Herein, we report two active-state cryo-EM structures of GPR55 in complex with a modified G_13_ protein using two different agonists: (1) LPI as one of the proposed endogenous ligands at 2.96 Å global resolution and (2) the synthetic agonist ML184 at 2.64 Å global resolution (for ligand structures see Fig. 1). Notably, our work delivers a structural understanding of the role of LPI as a putative endogenous activator of GPR55 and provides structural insights into the recognition of structurally diverse agonists at GPR55. Our results will be imperative for the structure-based development of pharmacological tool compounds and emerging drugs targeting GPR55.

## Results

### Architecture of the GPR55-Gα_13_β_1_γ_2_ Signaling Complex

The formation of a stable GPR55-G protein-ligand complex for cryo-EM was achieved by employing the mini-G protein strategy^43^ using a previously described Gγ_2_-mini-Gα_13_ and His-Gβ_1_ tandem vector^40^ in which mini-Gα_13_ is fused to the *C*-terminus of Gγ_2_. The initial 30 *N*-terminal residues of mini-Gα_13_ were replaced by amino acids of Gα_i2_^40^ to enable binding of a single chain variable fragment (ScFv16)^44^ for increased complex stability (Fig. 1). For simplicity, the G protein complex of Gγ_2_-mini-Gα_13/Ni2_ and His-Gβ_1_ is henceforth referred to as Gα_13_β_1_γ_2_. The structure of GPR55 was determined using the full-length, wild-type (wt) receptor with additions to its *N*- and *C*-terminus (see “Methods”). Importantly, the *N*-terminus was fused to a Green Fluorescent Protein (GFP) mutant with enhanced fluorescence and folding properties (Folding Reporter GFP)^45^, enabling the expression and purification of stable full-length GPR55 in complex with G proteins (Supplementary Fig. S1).

**Fig. 2:**
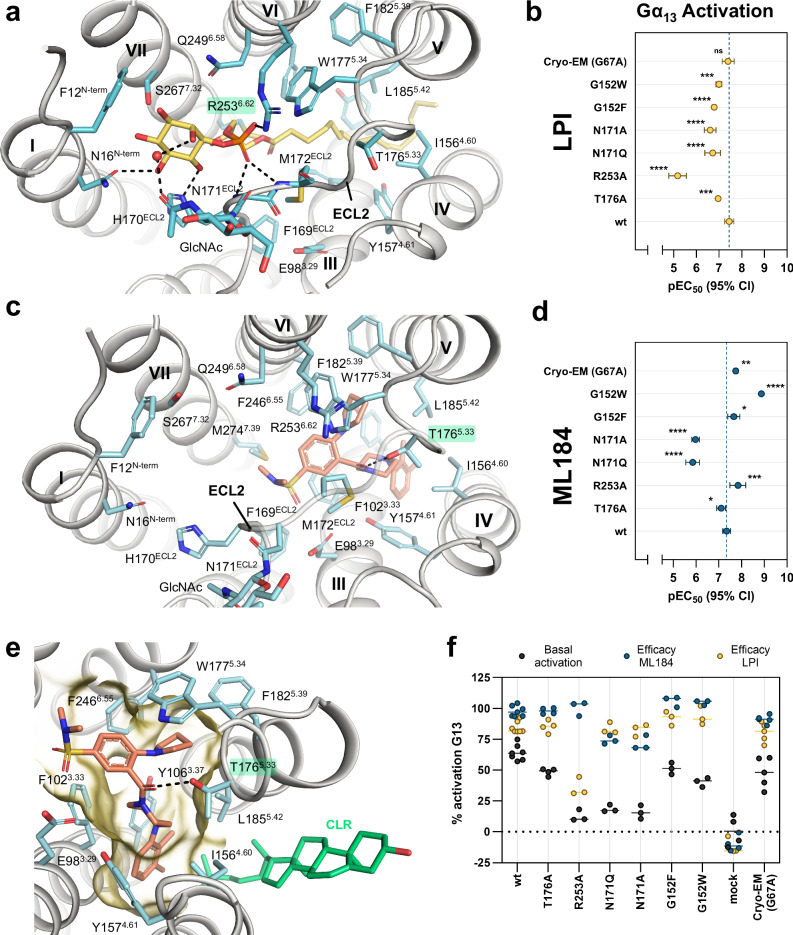
Agonist recognition at GPR55. **a** Ligand binding pocket of LPI (yellow sticks). The amino acid side chain and backbone that show interactions with LPI are shown as cyan sticks. Hydrogen bonds are indicated as black dashed lines. **b** Effect of GPR55 mutants on LPI potency as determined by G protein dissociation assays with Gα_13_. Data represent means ± 95% confidence interval (CI) from 3–7 independent experiments as indicated in Supplementary Table S3. **c** ligand binding pocket of ML184. **d** Effect of GPR55 mutants on ML184 potency as determined by G protein dissociation assays with Gα_13_. Data represent means ± 95% CI from 3–7 independent experiments as indicated in Supplementary Table S3. **e** Magnified view of the ML184 (salmon sticks) binding pocket (as in **a**). The binding pocket surface was displayed in yellow with the ECL2 surface hidden for better visibility of the binding pocket. **f** Efficacy and constitutive activity at different GPR55 mutants. The degree of activation was calculated by normalization of BRET² ratios (BRET² ratio of 30 µM ligand for efficacy or basal BRET² ratios of 1.5% DMSO) to the respective BRET² ratios for the wt GPR55 plus G_13_-biosensor at 30 µM ML184 (100% activation) and for a mock-transfection plus G_13_-biosensor (0% activation). The pEC_50_ means of all seven mutations were compared with the pEC_50_ mean of the wt GPR55 to evaluate statistically significant differences using ordinary one-way ANOVA with Dunnett’s post-hoc test (adjusted *P*-values: *p < 0.05, **p < 0.01, ***p < 0.001, ****p < 0.0001, for absolute *P*-values see Supplementary Table S3).

The GPR55-G protein complexes were determined by single-particle cryo-EM, resulting in three-dimensional (3D) cryo-EM maps for all components except GFP, which exhibited the anticipated flexibility (Fig. 1 and Supplementary Fig. S2). Both the GPR55-Gα_13_β_1_γ_2_-ScFv16-LPI and GPR55-ML184-Gα_13_β_1_γ_2_-ScFv16 complexes were reconstructed, resulting in a global resolution of 2.96 Å and 2.64 Å, respectively (Fourier shell correlation, FSC, of the consensus map at threshold 0.143), whereas the local refinement on the receptor resulted in 3.01 Å (GPR55-LPI) and 2.77 Å (GPR55-ML184) resolution. The local resolution of the orthosteric site was estimated to be 2.9 Å (LPI) and 2.6 Å (ML184), which allowed for unambiguous modeling of the binding pose for both ligands (Fig. 2 and Supplementary Fig. S3). Cryo-EM refinement and validation statistics are shown in Supplementary Table S2.

To validate the functionality of the GPR55 cryo-EM construct, we conducted bioluminescence resonance energy transfer 2 (BRET²)-based G protein dissociation assays^46,47^ using an engineered heterotrimeric G_13_ protein. The *N*-terminal GFP fusion of the receptor was additionally modified with a G67A mutation to disrupt fluorescence properties^48^. We employed human embryonic kidney (HEK) cells overexpressing wt or mutant GPR55 together with Gβ_3_, Gγ_9_-GFP, and Gα_13_ tagged with *Renilla* luciferase 8 (Rluc8). Hence, the dissociation of the Gα-subunit from the Gβγ-dimer upon activation results in decreasing BRET² responses. Both ligands, LPI and ML184, activated the cryo-EM construct (pEC_50_ 7.40 ± 0.06 and 7.74 ± 0.03) with similar properties compared to wt GPR55 (pEC_50_ 7.45 ± 0.08 and 7.35 ± 0.07) (Supplementary Table S3 and Supplementary Fig. S4), indicating that the cryo-EM construct is fully functional.

### The binding mode of the putative endogenous ligand elucidates crucial roles of R253^6.62^ and N-linked glycosylation at N171^ECL2^ in lipid recognition

The phosphate group of LPI is anchored at the extracellular ends of GPR55 by forming salt-bridge interactions to R253^6.62^ located in transmembrane helix VI (Fig. 2a) (superscript residue numbers refer to the Ballesteros-Weinstein numbering system^49^). The extracellular loop (ECL) 2 of GPR55 is folded into the receptor binding pocket and additionally stabilizes the phosphate by hydrogen bonding of the backbones of N171^ECL2^ and M172^ECL2^ (Fig. 2a). The importance of R253^6.62^ was further confirmed by evaluating a R253^6.62^A mutation in the G protein dissociation assay which drastically reduced the potency of LPI by 193-fold, compared to wt GPR55 (Fig. 2b, Supplementary Fig. S4, and Supplementary Table S3). In addition, the efficacy of LPI was lowered to 36%, and the basal activation of the R253^6.62^A mutant was determined to be only 13% compared to wt with 66% basal activation (Fig. 2f and Supplementary Table S3). Notably, for G protein dissociation assays with wt GPR55 and its mutants, we employed untagged native receptor sequences which prevented the quantification of their surface expression in HEK cells. We cannot exclude the possibility that differential receptor expression may potentially affect the comparison of ligand potency and efficacy as well as basal receptor activation.

The inositol head group of LPI is located at the extracellular surface of GPR55 (Fig. 1b and d) with contacts to the *N*-terminal end of helix I and to ECL2. Specifically, it forms direct hydrogen bond interactions to N16^N-term^ and H170^ECL2^ (Fig. 2a). The ECL2 of GPR55 features an N-linked glycosylation site (N171^ECL2^) of which the first *N*-acetylglucosamine (GlcNAc) was resolved (Fig. 2a and Supplementary Fig. S3). Interestingly, a water-mediated hydrogen bond of LPI’s inositol head group to the *N*-acetyl oxygen was observed. This region is located at the receptor surface showing lower resolution and possibly higher flexibility. Consequently, the structural water molecule could be transient with a high bulk water exchange rate. Mutation of N171^ECL2^ to A or Q prevents receptor glycosylation and, compared to wt GPR55, reduced the potency of LPI by 5.5- and 7-fold, respectively, (Fig. 2b and Supplementary Table S3) without affecting its efficacy (Fig. 2f). Notably, both mutations (N171^ECL2^Q and N171^ECL2^A) led to a significantly decreased constitutive receptor activity (19% and 16% basal activation, respectively) (Fig. 2f).

**Fig. 3:**
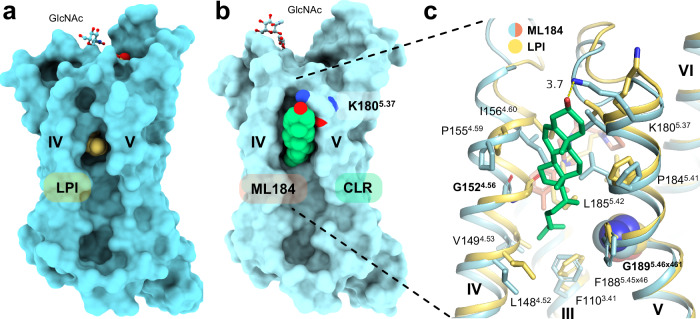
Membrane opening close to orthosteric pocket and cholesterol binding site. **a** Side view of the GPR55-LPI complex (cyan surface representation and yellow spheres, respectively) shows membrane gate into the hydrophobic channel of the orthosteric binding pocket between helices IV and V. Membrane lipids were not resolved in this structure. **b** Side view of the GPR55-ML184 (light cyan surface representation) complex resolved a CLR molecule (green spheres) bound in a similar cleft between helices IV and V. **c** Superimposition of the GPR55-LPI and GPR55-ML184 structures (receptor in cartoon representation, with side chains that contact CLR as sticks) show the CLR (green sticks) binding pocket with direct contacts to ML184 (salmon sticks). Subtle sidechain rearrangements in the LPI structure within the CLR interface are observed. The dotted yellow line represents the distance measurement (in Å) between K180^5.37^ and CLR.

The glycerol moiety of LPI is not involved in any specific hydrogen bond interactions. Although the 2-hydroxy group faces toward a receptor cavity mainly formed by Q23^1.35^, K80^2.60^, Q271^7.36^, and M274^7.39^ (Supplementary Fig. S5 and Fig. 1d), structural water molecules potentially interacting with the hydroxy group were not resolved. The adjacent lipophilic palmitoyl tail of LPI winds through a narrow channel within the receptor with van der Waals contacts to F102^3.33^, Y106^3.37^, S153^4.57^, I156^4.60^, Y157^4.61^, T176^5.33^, W177^5.34^, L185^5.42^, and F246^6.55^ (Figs. 1d,  2a and Supplementary Fig. S5). The cryo-EM map for the palmitoyl chain is not fully continuous at higher contour levels (Supplementary Fig. S3) and likely exhibits some residual flexibility when bound to the receptor. Therefore, the modeled binding mode of the chain represents the most plausible conformation in the binding pocket channel. The palmitoyl chain exits the hydrophobic channel via a membrane opening between helices IV and V where it might contact other membrane constituent lipids (Figs. 1d, 2a,  3a and Supplementary Fig. S5). The membrane opening is, in part, formed by two glycine residues (G152^4.56^ and G189^5.46×461^) that are located on opposite ends of the opening (Fig. 3c and Supplementary Fig. S5). Mutations of G152^4.56^ to larger amino acids (F and W) were designed to potentially reduce the size of the membrane opening and to evaluate its importance for LPI potency. The mutations reduced the potency of LPI by 3- to 5-fold (Fig. 2b) but did not affect efficacy or basal receptor activation (Fig. 2f and Supplementary Table S3), indicating that bulkier amino acids at this position can provide a significant steric hindrance without completely abolishing LPI activity.

### Structure with synthetic agonist ML184 reveals a ligand-cholesterol interaction

The synthetic agonist ML184 binds to the hydrophobic cavity that is populated by the palmitoyl tail in the LPI-bound structure. Structural rearrangements, like the outward movement of the extracellular end of helix V by 3.5 Å, are required to accommodate the sterically more demanding ligand in this pocket (Supplementary Fig. S5). Accordingly, the binding mode of the synthetic agonist ML184 is characterized by hydrophobic contacts and aromatic stacking interactions (Fig. 2c and e). The sulfonamide directly interacts with E98^3.29^, F102^3.33^, F169^ECL2^, M172^ECL2^, L270^7.35^, and M274^7.39^ (Fig. 2c and e). The phenyl ring adjacent to the sulfonamide forms multiple face-to-edge aromatic stacking interactions with F102^3.33^, W177^5.34^, as well as F246^6.55^ and is additionally stabilized by contacts to M172^ECL2^ and M274^7.39^ (Fig. 2c and e). Notably, the ECL2 between M172^ECL2^ and D175^ECL2^ as well as the adjacent R253^6.62^ sidechain, that is essential for GPR55 activation by LPI, appear to be more flexible in the ML184-structure (Supplementary Fig. S3). Direct interaction of R253^6.62^ with ML184 was not observed. However, R253^6.62^ is located ~ 8 Å from the sulfonamide moiety of ML184 and R253^6.62^ is accessible via a solvent channel that extends into the inositol pocket of LPI (Figs. 2c and  1g). Accordingly, the potency of ML184 at the receptor containing the R253^6.62^A mutant was not reduced but slightly increased by a factor of three with an efficacy similar to the wt receptor (Fig. 2d and f).

The pyrrolidine moiety of ML184 addresses a lipophilic sub-pocket formed by W177^5.34^, F182^5.39^, L185^5.42^, and F246^6.55^ (Fig. 2e). Notably, a previous structure-activity relationship study investigating compounds related to ML184 found that larger aromatic residues seem to be tolerated in this position^36^. The sidechain of T176^5.33^ adopts a different rotamer in the ML184 structure (rotated by approximately 90°) and forms a direct hydrogen bond with the amide carbonyl of ML184 (Fig. 2c and e). The mutant T176^5.33^A resulted in a 2-fold reduction of the ML184 potency and did not affect ligand efficacy (Fig. 2d and f and Supplementary Table S3), indicating that the hydrogen bond is not essential for the affinity of ML184. Interestingly, the same mutation reduced the potency of LPI by 3-fold (Fig. 2b). In the ML184 structure, N-linked glycosylation was also observed and, in this case, the first two GlcNAc glycans were resolved (Supplementary Fig. S6). However, interactions of N171^ECL2^ or its glycans with ML184 were not observed. Surprisingly, mutations of N171^ECL2^ to A or Q still significantly reduced the potency of ML184 by 24- and 32-fold (Fig. 2d).

The piperazine ring of ML184 forms van der Waals contacts to F102^3.33^, Y106^3.37^, I156^4.60^, Y157^4.61^, T176^5.33^, and L185^5.42^ (Fig. 2c and e). The terminal dimethylphenyl ring is stabilized by aromatic stacking interactions with Y106^3.37^ and Y157^4.61^ (Fig. 2e) and points towards the same membrane opening between helices IV and V as observed for the lipid tail in the LPI-bound structure (Figs. 1g, 2c and 3). Small and flexible *o*,*p*-disubstituted ML184-derivatives were demonstrated to be tolerated at this position whereas larger substituents or bicyclic systems reduced ligand potency^36^. Remarkably, the ML184-structure revealed density that can be attributed to a cholesterol (CLR) molecule blocking the membrane opening within a cleft formed by helices IV and V (Figs. 2e,  3b and c and Supplementary Figs. S3 and S7). Admittedly, the CLR content of the employed insect cell membranes is significantly lower than that of mammalian cells^50^ and the CLR dicarboxylic acid monoester, cholesteryl hemisuccinate (CHS), was employed during purification. Therefore, CHS may likely be bound to the receptor instead of CLR. However, the cryo-EM map does not provide additional evidence for the esterification (Supplementary Fig. S3), and consequently, CLR was modeled.

The CLR forms contacts to the dimethylphenyl moiety of ML184 and to F110^3.41^, L148^4.52^, V149^4.53^, I156^4.60^, K180^5.37^, P184^5.41^, and L185^5.42^ (Fig. 3c). The side chain ammonium group of K180^5.37^ may interact with the hydroxy head group of CLR and is at 3.7 Å distance in the current model. However, the K180^5.37^ sidechain was not fully resolved in the cryo-EM map (Supplementary Fig. S3). As discussed for LPI, the access to the membrane opening is enabled by two glycine residues (G152^4.56^ and G189^5.46×461^) that are located opposite to each other (Fig. 3c and Supplementary Fig. S5). We also investigated the effect of G152^4.56^ mutants on the Gα_13_ activation by ML184. Mutations of G152^4.56^ to the sterically more demanding amino acids F and W may potentially alter CLR binding (Supplementary Fig. S7). Surprisingly, the G152^4.56^W mutation increased the potency of ML184 by 34-fold whereas the potency remained unchanged at the G152^4.56^F mutant (Fig. 2e). This increase in potency may be a result of improved hydrophobic interactions between the tryptophan side chain and the dimethylphenyl moiety in ML184 (Supplementary Fig. S7). Although the larger tryptophan sidechain would likely clash with the observed binding mode of CLR (Supplementary Fig. S7), it is unclear whether these mutants affect CLR binding or whether CLR binding has functional implications at all.

**Fig. 4:**
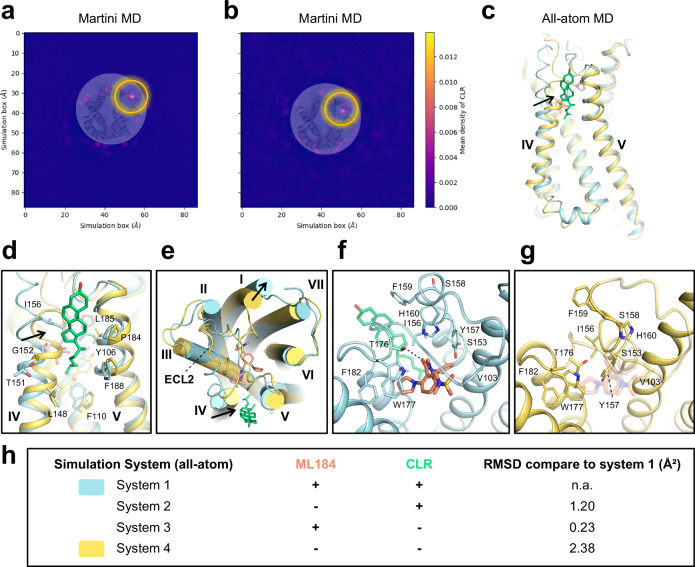
Molecular dynamics simulations investigate the CLR binding pocket stability. **a**, **b** Averaged CLR mass density plot generated from martini coarse-grained simulations for (**a**) martini system 1 (CLR is placed in the structure pose) and (**b**) for martini system 2 (CLR was removed), the yellow circle highlights the CLR density in both figures. **c** Superimposition of final structures from all-atom system 1 (shown in cyan) containing CLR and ML184 with system 4 (shown in yellow) where ML184 and CLR were removed shows that significant changes in the protein conformation is observed (black arrow) when the CLR molecule was removed from the binding site. The structural CLR molecule is shown in green and ML184 is shown in orange. **d** Enlarged view of the CLR binding pocket and (**e**) surface representation show that CLR binding site is occluded significantly. The all-atom simulation without ML184 and CLR also has an impact on the ligand binding pocket as shown in (**f**, **g**). **h** Overview of four simulated all-atom systems with the RMSD of the ML184 binding pocket calculated from the representative structure of the most populated cluster, compared to system 1. n.a.: not applicable.

To better understand the interactions and role of CLR on binding pocket stability, we employed molecular dynamics (MD) simulations. First, we utilized coarse-grained martini simulations to investigate the specificity of the CLR molecule towards the CLR binding site. The martini simulations were carried out in two different setups: (1) CLR in the binding pocket and (2) CLR removed from the CLR binding site with no CLR within at least 2 nm of the protein in the membrane. For the latter case, we applied positional restraints on the protein backbone to prevent significant structural changes in the protein, which might possibly impede any protein-CLR interactions. Both systems were simulated for 10 µs with three replicates each in a lipid bilayer composed of dioleoylphosphatidylcholine (DOPC) and CLR in a 70:30 ratio. The molecular trajectories were analyzed to track the position of CLR, using a density analysis on the CLR head group (ROH martini bead) projected on the x-y membrane plane. The density map results indicated that CLR remains in the identified CLR binding site in system 1 (Fig. 4a) for all three replicates. For system 2, where CLR was removed from the CLR binding pocket, we observed a CLR molecule diffusing towards the CLR binding site from the membrane within approximately 600 to 1300 ns. Within the next few hundred ns, the CLR inserted into the same CLR binding site (Fig. 4b).

We then performed all-atom MD simulations in four different setups to investigate the role of CLR for the structural stability of GPR55 (Fig. 4h). All simulations were conducted for 1 µs with three replicates, each in a hydrated lipid bilayer consisting of DOPC and CLR in a 70:30 ratio. The simulation trajectories were clustered based on protein backbone coordinates. The centroid structure from the largest cluster was selected for further analysis employing the root-mean-square deviation (RMSD) of the ML184 binding pocket amino acid residues from the cryo-EM structure compared to the representative structures after the simulation. We found that the setup in systems 1–3 (Fig. 4h), which included either both ligands, ML184 and CLR (system 1), or each of the ligands alone (system 2 and 3, respectively) in the simulation, had little to no effect on the conformation of the binding pocket, as the ligand binding pocket RMSD was <1.3 Å compared to the cryo-EM structure. However, when both ML184 and CLR were removed from the simulation (system 4), the agonist and the CLR binding pocket were partially occluded (Fig. 4c–g), as indicated by an RMSD of the ligand binding pocket amino acid residues compared to the cryo-EM structure of 2.38 Å (Fig. 4h). Overall, these results suggest that the CLR molecule plays an important role in stabilizing the conformation of the binding pocket.

### Activation of GPR55 and Gα_13_ coupling interface

The two structures of GPR55 were determined in the G protein-bound active state and are highly similar with an RMSD of 1.91 Å considering all atoms and 1.42 Å within the G protein heterotrimer. The common activation microswitches of GPR55 were found to contain features of an activated conformation^51^ (Supplementary Fig. S8). Their arrangement is virtually identical between both structures, but for clarity, only the higher resolution structure in complex with ML184 is shown. The classical activation motifs described for class A GPCRs are not fully conserved in GPR55^52^. For instance, instead of the CWxP, DRY, NPxxY, and PIF motifs, GPR55 carries SFxP, DRF, DVxxY, and PVF motifs. These microswitches are more conserved between GPR55 and GPR35 (Supplementary Fig. S8f) and the recently determined active state structure of GPR35 in complex with G_13_ was therefore used for comparison^41^. In addition, we compared GPR55 to an inactive state AlphaFold2 model^53,54^ of GPR55 (downloaded from GPCRdb^55^ and to inactive^56^ and active^57^ state structures of the prototypical β_2_ adrenergic receptor (β_2_AR) although the receptors do not share identical motifs (Supplementary Fig. S8f). Notably, both GPR55 and GPR35 exhibit virtually identical activated microswitch conformations, consistent with the active state of the β_2_AR. However, GPR55 and GPR35 contain the transmission switch residue F^6.48^ instead of W^6.48^ and only show small helix VI outward movements (4-5 Å) when compared to the large 14 Å switch of the β_2_AR (Supplementary Fig. S8). Within this comparison, the intracellular end of helix I shifts outward, and helix VII moves into the G protein binding site as similarly observed for the β_2_AR. In contrast, helix V of GPR55 and GPR35 is practically stationary during activation, whereas helix V of the β_2_AR shows an additional outward movement. The inactive state AlphaFold2 model of GPR55 is moderately similar to the active GPR55 cryo-EM structure (RMSD 3.3 Å) and aligns well with the overall conformation of the inactive β_2_AR (Supplementary Fig. S8), supporting the predicted conformational changes. These results indicate that the underlying activation mechanism of GPR55 (and GPR35^41^) seems to have features related to classical GPCR activation^51,52^, but also aspects that enable Gα_13_ binding without large helix V and VI outward movements. The determination of inactive state structures of the same receptor will be instrumental to further explore the mechanism of GPR55 activation.

**Fig. 5:**
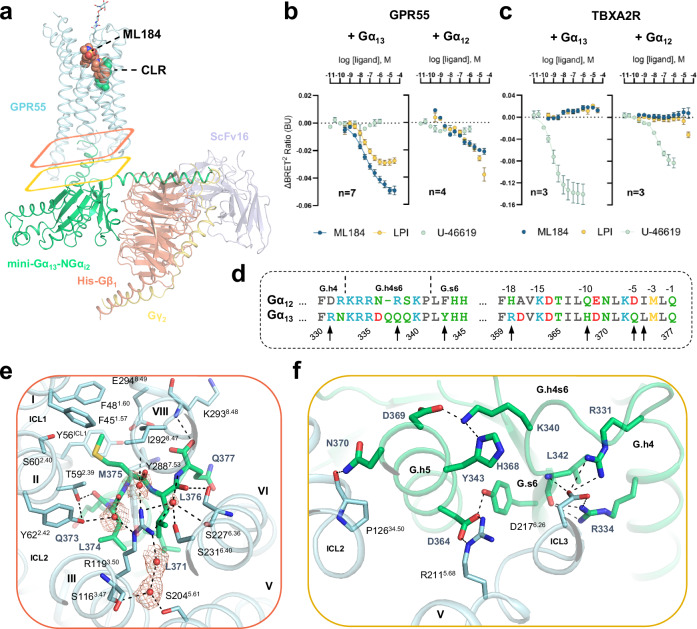
Gα_13_ activation by GPR55 and selectivity over Gα_12_. **a** Overview of the GPR55-G_13_-ML184 signaling complex. ML184 and CLR are shown as salmon and green spheres, respectively. Colored rectangles highlight specific G protein interaction sections as described in panels (**e**, **f**), respectively. The map quality of one water molecule deviates significantly from four well-resolved water molecules. The modeling of this water molecule was guided by its coordination with R119^3.50^, helix VI, and helix VII. **b**, **c** G protein dissociation assays of (**b**) GPR55 and (**c**) TBXA2R with Gα_12_ and Gα_13_. Assays were performed in HEK293H cells transiently transfected with wt Gβ_3_, Gγ_9_-GFP, and Gα_12_-Rluc8 or Gα_13_-Rluc8. Data represents means ± SEM from 3–7 independent experiments as specified in Supplementary Table S3. The negative control data for U-46619 at GPR55 and ML184 or LPI at the TBXA2R was analyzed from three independent experiments. **d** Sequence alignment of different G protein segments of Gα_13_ involved in GPR55 binding with the respective residues of Gα_12_. Black arrows highlight amino acid differences between Gα_12_ and Gα_13_ within 4 Å of GPR55. Residues R360 and Q338 are predominantly solvent-exposed and not shown in the following panels. **e** Protein-protein interface between Gα_13_ (green cartoon and sticks) and GPR55 (cyan cartoon and sticks) with a focus on the *C*-terminal α5-helix of Gα_13_. The cryo-EM composite map (EMDB-51284) for five water molecules is shown with orange mesh. **f** Protein-protein interactions of residues between the α4-helix and β6-sheet of Gα_13_ as well as of the initial α5-helix residues with GPR55.

Next, we analyzed the receptor-G protein interface focusing on the ML184-bound structure due to its higher resolution (Fig. 1 and Supplementary Fig. S2). Protein-protein interactions between GPCR and G proteins, as well as Gα selectivity are predominantly mediated through the *C*-terminal α5 helix of the G protein^58^. To enable a clear comparison between G proteins, the α5-helix was additionally labeled by superscript reverse numbering starting from the *C*-terminus. The Gα_12/13_-family stands out by having a *C*-terminal glutamine residue (Q377^-1^) where other G protein families feature hydrophobic amino acids. In GPR55, the carboxy terminus of Q377^-1^ forms a hydrogen bond to the backbone of K293^8.48^ (Fig. 5e). The methionine in position -3 (M375^-3^) represents another distinctive feature of the Gα_12/13_ family. Its side chain is positioned in a hydrophobic pocket formed by F45^1.57^, F48^1.60^, Y56^ICL1^, T59^2.39^, S60^2.40^, I292^8.47^, and E294^8.49^ (Fig. 5e). The arginine (R119^3.50^) of the conserved D^3.49^R^3.50^Y^3.51^ motif (DRF motif in GPR55) forms an ionic lock in many inactive state GPCRs^59^. In the active GPR55, the side chain of R119^3.50^ is involved in an extensive water network that connects the arginine to helices II (Y62^2.42^), III (S116^3.47^), IV (S204^5.61^), VI (S227^6.36^ and S231^6.40^), and VII (amide backbone of Y288^7.53^) (Fig. 5e). Interactions of R119^3.50^ with Gα_13_ are mediated through hydrogen bonding to the backbone amides of L374^-4^ and M375^-3^ via structural water molecules (Fig. 5e). Additional hydrogen bond interactions of GPR55 with the α5-helix were observed between T59^2.39^ and the backbone of Q373^-5^ as well as S277^6.36^ and the backbone of L376^-2^ (Fig. 5e). Apart from a tightly packed α5-helix, other structural elements of Gα_13_ interact with all GPR55 intracellular loops (ICLs) (Fig. 5e and f). Notably, Y56^ICL1^ of the ICL1 folds into the G protein binding site, representing a unique aspect of the GPR55-G protein interface. Moreover, the intracellular end of helix VI (D217^6.26^) is involved in unprecedented salt-bridge interactions with R331 and R334 of the α4β6-loop (Fig. 5f).

Guided by structural insights, we employed the G protein dissociation assay to validate the G protein selectivity of GPR55. The sequences of Gα_13_ and Gα_12_ are highly similar (76% sequence similarity) and only seven Gα_13_ residues differ within a 4 Å sphere of the receptor- G protein interaction site (Fig. 5d). Therefore, we included Gα_12_ in our assays as a control since it was reported that 1-AG-PI-activated GPR55 signals exclusively via Gα_13_^10^. Herein, we observed robust G protein activation (concluded from a BRET² ratio decrease) when GPR55 was co-expressed with Gα_13_ (Fig. 5b), even without the addition of any ligand. (Fig. 2f and Supplementary Table S3). This observation is indicative of constitutive activity; however, it could also be explained by GPR55 interaction with cellular lipids that are ubiquitously present. Both ligands, LPI and ML184, induced Gα_13_ activation in a concentration-dependent manner and showed similar potency (EC_50_ 35.5 nM and 44.7 nM, ΔpEC_50_ 0.1, *P* = 0.3654, unpaired *t* test). The efficacy of LPI was lower than that of ML184 (Figs. 2f and 5b and Supplementary Table S3), possibly a result of poor LPI solubility. When GPR55 was co-expressed with Gα_12_, a BRET² ratio decrease was observed at high ligand concentrations (Fig. 5b) but not in the absence of ligand (Supplementary Table S3). However, the data did not allow for a robust sigmoidal curve fit and EC_50_ value determination, indicating poor coupling efficiency. As a positive control, we employed the thromboxane A2 receptor (TBXA2R) together with the agonist U-46619^60^ due to its ability to activate both Gα_12_ and Gα_13_ with high potency^10,61^ (Fig. 5c). Herein, the potency of U-46619 at the TBXA2R was determined to be 24.0 nM and 0.93 nM via Gα_12_ and Gα_13_, respectively (Fig. 5c and Supplementary Table S3). Both GPR55 ligands showed no effect on TBXA2R-transfected cells and, vice versa, U-46619 was inactive at GPR55-transfected cells (Fig. 5b and c and Supplementary Fig. S4).

Next, we used a mutagenesis approach to further explore the Gα_13_ selectivity of GPR55. The striking difference in the sequence between the two G protein subtypes guided the design of four Gα_13_ mutants that are in close contact with GPR55 by replacing the residues with the respective Gα_12_ amino acid (L374^-4^I, Q373^-5^D, H368^-10^Q, R331D). The mutants were tested individually for their ability to be activated by GPR55 using the same G protein dissociation assay. Three of the mutants (L374^-4^I, Q373^-5^D, and R331D) displayed reduced basal activation compared to wt Gα_13_ by approximately 2- to 4-fold whereas the basal activation of H368^-10^Q was nearly identical to that of wt Gα_13_ (Supplementary Table S3). The single Gα_13_-mutants affected the potency of LPI and ML184 only to a minor extend compared to wt Gα_13_ (Supplementary Table S3).

## Discussion

This work presents high-resolution structures of the orphan lipid-activated GPCR GPR55. The structures were determined via cryo-EM in complex with the effector heterotrimeric G protein G_13_ and structurally diverse ligands: (1) the putative endogenous agonist LPI and (2) the synthetic agonist ML184. The structure of LPI can be divided into three chemically distinct sections that shape its recognition at GPR55: the hydrophilic phosphoinositol head group, the glycerol moiety, and the lipophilic palmitoyl tail. The inositol moiety binds in a pocket close to the extracellular receptor surface with multiple hydrogen bond interactions. Remarkably, the first GlcNAc moiety N-linked to N171^ECL2^ is located near the inositol and forms a hydrogen bond interaction to LPI via one structural water molecule. In the ML184-structure, two connecting GlcNAc units were resolved but without showing direct ligand interactions. N-linked glycosylation at the ECL2 represents a common post-translational modification of GPCRs^62,63^, but their glycans are rarely visible in experimental GPCR structures (see Supplementary Table S4 for an overview) or glycosylation is removed prior to structural studies^64^. For example, in class A GPCRs, ECL2 N-linked glycosylation was resolved in structures of the serotonin 5-HT_2B_^65^ and angiotensin II receptors^66^ but without ligand interactions (Supplementary Table S4). We discovered that intact receptor glycosylation is essential for basal GPR55 activation and ligand potency, even though ML184 did not show direct glycan interactions. Therefore, we hypothesize that the removal of the ECL2 glycosylation site may affect the structural integrity or dynamics of the ECL2 which, in consequence, could explain the reduced activation with and without ligands.

The recognition of endogenous lysophospholipids by their respective GPCRs is typically mediated through interactions of negatively charged phosphate groups to either arginine or lysine side chains (Supplementary Fig. S9). High-resolution structures of several GPCRs with endogenous lysophospholipid agonists have been determined and revealed the binding mode of lysophosphatidylcholine (LPC) at GPR34^67^, lysophosphatidylserine (LPS) at GPR119^68^ and GPR174^69^, S1P at the S1PR_1-3_^42,70,71^, and LPA at the LPAR_1_^70^ (Supplementary Fig. S9). In the case of GPR55, salt-bridge interactions of LPI with R253^6.62^ of helix VI were observed. In contrast, diverse interactions of the phosphates of LPC, LPS, LPA, and S1P were observed with residues located in helices II^69^, III^70^, IV^69^, VII^68^, the ECL2^67^, or at the *N*-terminus^42,70,71^ of their GPCRs (Supplementary Fig. S9). Therefore, the location of the salt-bridge interaction partner of the negatively charged phosphate group is highly receptor-specific and does not seem to follow a general pattern for structurally similar lysophospholipids. The mutations R253^6.62^A and N171^ECL2^Q drastically reduced both receptor basal activation and ligand potency of LPI at GPR55. Recent studies on the identification of oleic acid as an endogenous ligand for GPR3^72–74^ provided an alternative hypothesis to explain the reported constitutive receptor activity. It was discussed that receptor activation could also be a result of the high population of oleic acid, as ubiquitous membrane component, in the binding site, leading to receptor activation instead of constitutive activity mediated by ligand-free GPR3. As phosphoinositol-containing lipids are widely prevalent as signaling lipids in the cytoplasmic leaflet^30,75^, we cannot exclude the possibility of a similar mechanism of GPR55 activation by LPI. Accordingly, the strong reduction in constitutive activity of the R253^6.62^ and N171^ECL2^ mutants could also be explained by a loss of potency of endogenous lipids. Admittedly, even single point mutations can affect the receptor’s surface expression, and differential receptor expression may potentially influence the comparison of ligand potency and efficacy or the basal receptor activation. However, none of the investigated GPR55 mutations was completely inactive, and some mutations (e.g., R253^6.62^A or G152^4.56^W) even showed opposite effects on the potency of both ligands which cannot be explained solely by altered receptor expression.

The glycerol moiety of LPI did not show polar interactions within the binding pocket. However, the glycerol is loosely packed and exposed toward a receptor cavity where it could potentially form water-mediated interactions. The lipophilic palmitoyl tail of LPI extends through a lipophilic pocket and exits the receptor via a membrane opening between helices IV and V. This opening may potentially function as an entrance and exit route for LPI to and from the orthosteric binding pocket, e.g., via lateral diffusion within the membrane. Considering the amphiphilic nature of LPI, access from the membrane via this route would likely entail larger re-arrangements of helices IV and V as well as ECL2 to facilitate lateral movement of the lipid tail and the polar phosphoinositol headgroup along the axis of the membrane surface. Notably, the abovementioned receptors for the lysophospholipids LPC, LPS, and S1P exhibited similar membrane openings between helices IV and V (Supplementary Fig. S9)^42,67–71^. Recently, the same observation was also made for oleic acid at GPR3^72–74^ and for the synthetic cysteinyl leukotriene receptor antagonist zafirlukast^76^. For both receptors, a similar access route for ligands to the binding pocket from the membrane was hypothesized^72,76^. In contrast, the LPAR1 does not show an opening to the membrane and the lipophilic tail of LPA folds back into the receptor^70^. In conclusion, the general location of the membrane opening proves to be somewhat conserved between helices IV and V in thus far determined lipid receptor structures. It remains to be investigated whether lipidic ligands use the membrane opening between helices IV and V as an entrance route to access the binding pocket.

In this study, a natural product mixture of lysophosphatidylinositols containing different fatty acid substitutions derived from soybeans were used. The 1-palmitoyl-substituted LPI represents the main constituent^29^ and was modeled in the cryo-EM structure based on the density map. However, we cannot exclude the possibility that a differentially substituted LPI species is present in the complex. For instance, longer lipophilic moieties would possibly extend through the membrane opening into the membrane and would likely not be fully resolved due to their flexibility. The membrane opening was also found in the ML184-bound structure. In this case, however, we observed a CLR membrane lipid bound to a cleft near the membrane opening. CLR directly interacts with ML184 and sterically blocks the opening to the membrane. The mutant G152^4.56^W within the entrance of the opening may potentially alter CLR binding. Interestingly, our results show a significantly higher potency of ML184 at this mutant, whereas G152^4.56^F did not affect ML184 potency. This increase in potency may result from additional direct interactions of the tryptophan side chain with ML184. A model of the mutations in the ML184-structure confirmed that both mutations may directly contact ML184 (Supplementary Fig. S7). Furthermore, G152^4.56^W, in contrast to G152^4.56^F, is capable of fully closing off the membrane opening. Therefore, G152^4.56^W may engage ML184 in a more favorable way than G152^4.56^F or CLR. On the other hand, the unaltered potency of ML184 at the G152^4.56^F mutation indicates that the phenylalanine can compensate for a potential loss of CLR interactions or that CLR is not vital for ML184 potency. Interestingly, our MD simulation studies indicate a potential role of CLR in stabilizing the extracellular receptor portion around the membrane opening and the ML184 binding pocket in the absence of an orthosteric ligand. Relatedly, in MD simulations of the cysteinyl leukotriene receptor 1, it was previously demonstrated that the presence of an orthosteric ligand is essential to prevent the collapse of a similar membrane opening between helices IV and V^76^. CLR is often resolved in membrane regions of GPCR structures and has been described as a modulator of GPCR function^77^ while direct contacts of CLR with orthosteric GPCR ligands have not been observed.

The binding mode of ML184 is predominantly mediated by contacts to hydrophobic and aromatic amino acids. Remarkably, we discovered a single hydrogen bond interaction between the amide oxygen and the side chain of T176^5.33^. However, a corresponding T176^5.33^A mutation affected the potency of ML184 only to a minor extent. Previous docking experiments^32^ have attempted to estimate the binding mode of ML184 using a GPR55 homology model and suggested hydrogen bonding interactions to K80^2.60^ and Q249^6.58^ by the sulfonamide oxygens and the pyrrolidine nitrogen of ML184, respectively. In the cryo-EM structure, these residues are located 5.5 to 7 Å from the ligand, and therefore, the predictions were not confirmed. The binding pocket of ML184 is populated by the palmitoyl tail in the LPI-bound structure (Supplementary Fig. S5). The corresponding inositol binding pocket is located towards the extracellular end close to ECL2 (Fig. 1, Supplementary Fig. S5) and is not addressed by ML184. Polar modifications of the synthetic agonist that extend toward the phosphoinositol pocket may allow to improve solubility and selectivity while maintaining or even improving potency.

Using different cannabinoid ligands and 1-AG-PI, GPR55 has been reported to canonically signal via Gα_13_ proteins exclusively^10,11^. In our hands, GPR55 also showed a distinct preference for Gα_13_ over the closely related G protein Gα_12_. So far, structures of only two class A GPCRs (GPR35 and S1PR_2_) have been determined in complex with G_13_^41,42^. Both receptors are less restrictive in their G protein interaction and can also activate, for instance, Gα_12_^10^. In comparison with these structures, M375^-3^ of the Gα_13_ α5-helix is more tightly packed by residues in GPR55 and may represent an important selectivity determinant over other G protein families that have different residues in position -3^41^. In the corresponding GPR35 or S1PR_2_ G protein complex, M375^-3^ is embedded in a similar hydrophobic environment. However, I^8.47^ is exchanged for the smaller A^8.47^ in the case of GPR35 whereas helix VIII was not resolved in the S1PR_2_-structure. To probe the G protein selectivity of GPR55, we employed four Gα_13_ mutants located in the GPR55-G protein interface. Three mutations (L374^-4^I, Q373^-5^D, and R331D) displayed reduced basal activation with little to no impact on ligand potency. One Gα_13_-mutant (H368^-10^Q) showed no effect on ligand potency or basal activation for either agonist, indicating that this residue is less important for Gα_13_ selectivity.

In summary, two high-resolution GPR55 structures were determined in complex with the relevant effector protein. The structures reveal the so far elusive binding modes of the putative endogenous ligand as well as a synthetic GPR55 agonist and greatly advance our understanding of GPCR-G protein interactions of the understudied Gα_12/13_ family. The findings presented herein will accelerate structure-based design campaigns to develop pharmacological tools and drugs targeting GPR55.

## Methods

### Design and expression of the GPR55-Gα_13_β_1_γ_2_-ScFv16 complex

The GPR55 cryo-EM construct representing the full-length wt receptor with *N*- and *C*-terminal additions was discovered during a construct screening campaign at Nuvisan GmbH (Berlin, Germany). Nuvisan produced the baculovirus in *Spodoptera frugiperda* (*Sf9*) cells using the flashBAC expression system (Oxford Expression Technologies) with the baculovirus transfer vector pVL1393 as previously described^78^. The *N*-terminus of GPR55 was fused to a hemagglutinin signal sequence^79^, a FLAG® tag, and Folding Reporter GFP^45^ (residues 3-238, contains six point mutations: F64L, S65T, Q80R, F99S, M153T, and V163A), followed by a human rhinovirus 3 C protease cleavage site that is flanked by two linkers (*N*-terminal AAGSGEF and *C*-terminal GAGSDS). The *C*-terminus of GPR55 is followed by a linker (GAGSGAGS), a Streptavidin tag (Twin-Strep-tag®), another linker (GAGS), and a 10-fold histidine tag. The mini-Gα_13_-Gγ_2_ and His-Gβ_1_ tandem vector was designed as previously described^40^. Its coding sequences were codon-optimized for *Trichoplusia ni* (*Tni*) expression, gene-synthesized, and sub-cloned into the pFastBac^TM^-Dual vector (Thermo Fisher Scientific) between BamHI and EcoRI (His-Gβ_1_) as well as XhoI and KpnI (mini-Gα_13_-Gγ_2_) by GeneArt (Thermo Fisher Scientific). The Bac-to-Bac^TM^ expression system (Thermo Fisher Scientific) was used to produce the recombinant baculoviruses for the G proteins in *Sf9* insect cells (Thermo Fisher Scientific) according to the manufacturer’s instructions. Baculoviruses after three rounds of virus amplification (P3 virus) were used in a 1:1 ratio to express the GPR55-Gα_13_β_1_γ_2_ complex in *Tni* cells (HighFive^TM^, Thermo Fisher Scientific) at 1 ∙ 10^6^ cells mL^-1^ density using SF-900^TM^ II serum-free medium (Thermo Fisher Scientific). Successful expression of the GPR55-G protein complex components in insect cells was determined by western blotting using anti-penta-His (Thermo Fisher Scientific, cat. #P-21315, 1:1000 dilution) and anti-Gα_13_ (ABclonal, cat. #A20908, 1:2000 dilution) antibodies (Supplementary Fig. S1). Sodium dodecyl sulfate-polyacrylamide gel electrophoresis (SDS-PAGE) was performed with NuPAGE^TM^ bis-Tris (2-[bis(2-hydroxyethyl)amino]-2-(hydroxymethyl)propane-1,3-diol) 4–12% gradient gels (Thermo Fisher Scientific, cat. #NP0321) using 2-(*N*-morpholino)ethanesulfonic acid (MES) running buffer (Thermo Fisher Scientific, cat. #NP0002) and Precision Plus Protein^TM^ dual color standard (BioRad). Proteins were either stained with quick coomassie stain (ProteinArk) or blotted on nitrocellulose membranes with the iBlot^TM^ 2 dry blotting system (Thermo Fisher Scientific, cat. #IB21001). Blots were processed with an automated western blot processor (Bandmate^TM^, Thermo Fisher Scientific) using 3% bovine serum albumin (BSA) as a blocking agent and buffer [50 mM Tris (2-amino-2-(hydroxymethyl)propane-1,3-diol) pH 7.4, 150 mM NaCl, 0.005% polysorbate 20] for repeated washing. Alkaline phosphate-conjugated secondary antibodies (Sigma, cat. #A4312 or cat. #A3687, 1:30000 dilutions) were employed and detected with nitro blue tetrazolium chloride (NBT) and 5-bromo-4-chloro-1*H*-indol-3-yl dihydrogen phosphate (BCIP) using 1-Step^TM^ NBT/BCIP solution (Thermo Fisher Scientific, cat. #34042).

### Purification of GPR55-Gα_13_β_1_γ_2_-ScFv16-ligand complexes

Insect cells expressing the *N*- and *C*-terminally modified GPR55 and Gα_13_β_1_γ_2_ were lysed by osmotic shock in buffer [10 mM 2-[4-(2-hydroxyethyl)piperazin-1-yl]ethane-1-sulfonic acid (HEPES) pH 7.5, 10 mM MgCl_2_, 20 mM KCl], supplemented with cOmplete^TM^ ULTRA protease inhibitors (Sigma, cat. #6538282001) and DNAse I (Sigma, cat. #10104159001). The stable GPR55-Gα_13_β_1_γ_2_ complex was formed by the addition of 25 mU mL^−1^ apyrase (NEB, cat. #M0398) and agonists (10 µM LPI or 20 µM ML184), followed by incubation for 1.5 h at room temperature. LPI was obtained as a sodium salt from soy (Sigma, cat. #440153), and stock solutions at 10 mM were prepared in dimethyl sulfoxide (DMSO). ML184 was obtained from ArZa Bioscience (cat. #ARZ-EA084287) and stock solutions were prepared at 50 mM in DMSO. The complexes were solubilized directly from the lysates using equal volumes of solubilization buffer, resulting in the following buffer composition: 55 mM HEPES pH 7.5, 150 mM NaCl, 5 mM MgCl_2_, 10 mM KCl, 1% LMNG (2,2-didecylpropane-1,3-bis-β-d-maltopyranoside, Anatrace, cat. #NG310), 0.2% CHS-Tris (Anatrace, cat. #CH210), 10% glycerol, and 5 µM LPI or 10 µM ML184. After 1 h incubation at 4 °C, the suspensions were cleared by centrifugation at 100,000 x g (Beckmann Optima XPN-90). The pooled supernatant was incubated with 10 µL StrepTactin® XT 4flow® high-capacity resin (iba lifesciences, cat. #2-5030-025) per mL lysate for 2 h, agitated at 4 °C. The resin was transferred to empty gravity flow columns (BioRad) and washed with 20 column volumes of wash buffer (50 mM HEPES pH 7.5, 150 mM NaCl, 0.01% LMNG, 0.002% CHS-Tris, 10% glycerol, and 2 µM LPI or 5 µM ML184). Then, the GPR55-Gα_13_β_1_γ_2_ complex was eluted using five repeats of one column volume of elution buffer (25 mM HEPES pH 7.5, 150 mM NaCl, 0.003% LMNG, 0.0006% CHS-Tris, 10% glycerol, 50 mM biotin (Sigma, cat. #B4501), and 5 µM LPI or 5 µM ML184). The purified complex was supplemented with approximately 1.5 molar access of ScFv16 and incubated overnight at 4 °C. ScFv16 was produced by Selvita S.A. (Kraków, Poland) from *Tni* insect cells according to previously described methodology^44^. The following day, the GPR55-Gα_13_β_1_γ_2_-ScFv16 complex was subjected to size-exclusion chromatography using a Superose^TM^ 6 Increase 10/300 GL column (Cytiva, cat. # 29091596) on an ÄKTA pure^TM^ chromatography system (Cytiva) with the following running buffer: 25 mM HEPES pH 7.5, 150 mM NaCl, 0.003% LMNG, 0.0006% CHS-Tris, and 10 µM LPI or 10 µM ML184. Here, LPI was directly added to the buffer in solid form to avoid excess DMSO. Monomeric fractions that showed GFP absorption at 488 nm (see Supplementary Fig. S1 for chromatograms) contained all components of the GPR55-Gα_13_β_1_γ_2_-ScFv16 complexes were pooled and concentrated to ~7.5 mg mL^−1^ (LPI-complex) or 10 mg mL^−1^ (ML184-complex) using 50 kDa molecular weight cut-off Amicon® Ultra centrifugal filters (Merck).

### Cryo-EM sample preparation and image acquisition

Freshly prepared proteins were blotted on glow-discharged Quantifoil^TM^ holey carbon grids (R2/1, 300 mesh gold, Quantifoil Micro Tools) using a Vitrobot Mark IV system (Thermo Fisher Scientific). Grids were prepared at 4 °C and 70% humidity with Tedpella blotting paper (Thermo Fisher Scientific) and a blot force of -5 for 1.5 s, 40 s after sample application. Samples were vitrified by plunging into liquid ethane. Cryo-EM data for the LPI-complex was collected at the Research Institute of Molecular Pathology, IMP, Vienna, Austria, and the data for the ML184-complex was collected at the electron Bio-Imaging Center (eBIC), Diamond Light Source, Oxford, United Kingdom. Dose-fractionated movie frames were recorded on a Titan Krios^TM^ G4 cryo-transmission electron microscope according to Supplementary Table S2.

### Cryo-EM data processing

Data processing was performed with CryoSPARC (Structura Biotechnology), and an overview is provided in Supplementary Fig. S2. In summary, a total of 25,559 or 16,806 micrographs were collected for the LPI- and ML184-complexes, respectively. The micrographs were subjected to dose-weighting, beam-induced motion correction (patch motion-correction), and contrast transfer function (CTF) estimation (patch CTF). Then, blob-based particle picking was applied, and 5,188,047 particles (LPI-complex) or 13,541,164 particles (ML184-complex) were extracted using a box size of 300 × 300 pixels (binned to 100 × 100 pixels). Iterative rounds of 2D classification were used to polish the initial particle stack. Ab initio 3D reconstructions of particle subsets were used to refine the whole particle stacks using multiple rounds of heterogeneous refinement. Final 3D reconstructions of the full GPR55-Gα_13_β_1_γ_2_-ScFv16 complexes were generated via non-uniform refinement using 397,853 particles (LPI-complex) or 829,193 re-extracted particles (ML184-complex). Next, local refinements were carried out using a mask with the shape of the micelle which was generated from 3D reconstructions of the same dataset using ChimeraX^80^ and CryoSPARC. The final high-resolution structures of the GPCR alone were obtained by an additional local refinement using an additional mask with the shape of the GPCR. Composite maps were generated by combining consensus maps and local refined maps of the receptor with local refined maps of the Gα_13_β_1_γ_2_-ScFv16 complex using Phenix combine^81^. The cryo-EM maps of the full complexes (composite), the consensus maps, and the local refined maps have been deposited in the Electron Microscopy Data Bank (EMDB) with access numbers EMD-51288 (composite), EMD-51285 (consensus), EMD-51286 (focused map GPCR), and EMD-51287 (focused map G protein) for the GPR55-Gα_13_β_1_γ_2_-ScFv16-LPI complex and EMD-51284 (composite), EMD-51281 (consensus), EMD-51282 (focused map GPCR), and EMD-51283 (focused map G protein) for the GPR55-Gα_13_β_1_γ_2_-ScFv16-ML184.

### Model building and refinement

The AlphaFold2 model^53^ of GPR55 and the Gα_13_β_1_γ_2_-ScFv16 complex [from Protein Data Bank (PDB) ID 7YDH^82^] were fitted into the cryo-EM consensus maps (EMDB-51285 and EMDB-51281) as an initial model using ChimeraX^80^. Iterative cycles of model building via Coot^83^ and real-space refinement in Phenix^84^ using the final composite maps (EMDB-51288 and EMDB-51284) were performed to improve the model. Stereochemical restraints for LPI and ML184 were generated with Grade 2 (Global Phasing)^85^. Molprobity^86^ was used to guide the refinement process. Model refinement and validation statistics are presented in Supplementary Table S2. Structure figures were created with PyMOL (Schrödinger) or ChimeraX^80^. The structure coordinates of the GPR55-Gα_13_β_1_γ_2_-ScFv16-LPI and GPR55-Gα_13_β_1_γ_2_-ScFv16-ML184 complexes were deposited to the PDB with accession codes 9GE3 and 9GE2, respectively.

### G protein dissociation assays

G protein dissociation assays were performed according to the original protocols of the TRUPATH BRET² assay^47,87^ but with subtle modifications as previously described^61,88,89^. All reagents and cells were obtained from Thermo Fisher Scientific unless indicated otherwise. The coding sequences for the G protein biosensors^47^ (Gα_12_-Rluc8, Gα_13_-Rluc8, wt Gβ_3_, and Gγ_9_-GFP), the wt GPR55, the construct used for cryo-EM (with GFP bearing a G67A mutation to disrupt fluorescence properties^48^ for BRET² assays), GPR55 binding pocket mutants (G152^4.56^F, G152^4.56^W, N171^ECL2^Q, N171^ECL2^A, T176^5.33^A, R253^6.62^A), and the wt TBXA2R were codon-optimized for *Homo sapiens*, gene-synthesized, and sub-cloned into pcDNA^TM^3.1(+) between NheI and HindIII restriction sites by GeneArt (Thermo Fisher Scientific). The TBXA2R agonist U-46619 was obtained from Sigma (cat. #D8174). Adherent HEK293H cells were purchased from ThermoFisher Scientific and cultured in T175 flasks at 37 °C with 5% CO_2_ using Dulbecco’s Modified Eagle Medium (DMEM, cat. #11995073), supplemented with 2 mM l-glutamine, 10% fetal bovine serum, 100 U mL^-1^ penicillin, and 100 µg mL^−1^ streptomycin (cat. #25030024, #26400044, and #15140122, respectively). Freshly grown cells were washed with Dulbecco’s Balanced Salt Solution (DPBS, cat. #14200075) and detached using trypsin-ethylenediaminetetraacetic acid (trypsin-EDTA) (cat. #25300-062). Then, the cells were reconstituted to 500,000 cells per mL with fresh medium, and 1,000,000 cells were seeded into each well of a 6-well plate. After 3 h incubation at 37 °C and 5% CO_2_, adherent cells were transfected with 600 ng DNA (1:1:1:1 ratio of plasmid DNA for GPR55, wt Gβ_3_, Gγ_9_-GFP, and Gα_12_-Rluc8 or Gα_13_-Rluc8). Plasmid DNA was mixed with 1.5 µL Lipofectamine^TM^ 2000 (cat. #11668027) in Opti-MEM^TM^ medium (cat. #31985062) and incubated for 20 min. Then, the mixture was added to each well of the 6-well plate following 24 h incubation at 37 °C and 5% CO_2_. Transfected cells were washed with DPBS, detached using EDTA (Versene, cat. #15040066), and reconstituted into fresh medium at 500,000 cells mL^−1^. From this suspension, 50,000 cells were seeded into each well of a white-bottom 96-well plate (Nunc^TM^ MicroWell^TM^ 96 wells, cat. #136101) following overnight incubation at 37 °C and 5% CO_2_. Then, the adherent cells were washed with assay medium [Hank’s balanced salt solution (HBSS, cat. #14065056) plus 20 mM HEPES pH 7.4 (cat. #15630056)] and finally reconstituted into 50 µL of the same medium. Agonist stock solutions were prepared in DMSO at 10 mM (ML184) and 2 mM (LPI and U-46619). Agonist dilutions were prepared in assay buffer supplemented with 0.1% BSA for increased compound solubility. Methoxy e-Coelenterazine was used as luciferase substrate (Prolume Purple, NanoLight Technology, cat. #369), reconstituted at 1 mM in methanol and diluted to 37.5 µM with assay buffer (7.5 µM final assay concentration). The assay was started by the addition of 20 µL luciferase substrate to each well of the assay plate using a Multidrop^TM^ Combi reagent dispenser (Thermo Fisher Scientific), followed by 5 min incubation at room temperature. Then, 30 µL of agonist dilutions or DMSO control was added (final DMSO concentration 1.5%), followed by an additional 5 min incubation at room temperature. BRET² measurements were performed with a PHERAstar® FSX microplate reader (BMG LABTECH) using a BRET² filter (donor emission 370–450 nm, acceptor emission 500–530 nm) at the maximum fluorescence gain of 3600. BRET² ratios were calculated by dividing GFP emission signals from Rluc8 emission counts. Buffer-DMSO controls were subtracted from each data point to calculate ΔBRET² ratios. The half-maximal effective concentration (potency, EC_50_) and maximal effect (efficacy, E_max_) were determined by sigmoidal dose-response curve fit with variable slope. In addition, to calculate ligand efficacy and constitutive activity for each construct, the raw BRET ratios of their maximal ligand responses (at 30 µM ML184 or 30 µM LPI) and DMSO controls were normalized to the BRET ratios for a mock-transfected control at 1.5% DMSO comprising empty pcDNA^TM^3.1( + ), Gα_13_-Rluc8, wt Gβ_3_, and Gγ_9_-GFP (mock plus G_13_-biosensor, 0% activation) and for the wt GPR55 plus Gα_13_ biosensor (at 30 µM ML184, 100% activation). Data analysis was performed using GraphPad Prism 10 (GraphPad Software). Data was obtained in at least three independent experiments performed in duplicate.

### Martini molecular dynamics simulations

To evaluate the CLR-protein interaction at longer time scales, we employed coarse-graining with martini forcefield. The coarse-grained structure and topology for the protein was obtained with the martinize tool^90^, and was then embedded in a DOPC:CLR bilayer (70:30 ratio) with the insane tool^91^. The counter ions were added to neutralize the net charge of the simulation system. Two simulation systems were constructed as follows: martini system 1 with the structural CLR bound to the protein and martini system 2 with the CLR molecule removed from the system. In addition in system 2, it was ensured that no CLR molecule is present within 2 nm of the protein. All simulations were carried out using the GROMACS MD package^92^. The simulation system was hydrated with martini water particles. The assembled systems were then equilibrated for 50 ns with positional restraints applied to the protein beads with a force constant of 5 kcal per mol per Å^2^, followed by 100 μs of simulations in three replicates. The first 5 µs were treated as an equilibration run, and all analyses were performed in trajectories of 90 µs. The area per lipid property was used to determine the length of equilibration. During this production run for system 2, the force constant of 1 kcal per mol per Å^2^ was applied to the protein backbone. This was done to ensure that large structural changes in the protein could be avoided without having CLR bound in the binding pocket. The simulations were then analyzed for the averaged partial mass density of the CLR head group (ROH martini bead) with the MDAnalysis package^93^.

### All-atom molecular dynamics simulations

The initial structural conformation for the MD simulations was obtained from the ML184-bound structure, where in chain R, ML184 and CLR molecules were retained. Four simulation systems were constructed according to Fig. 4h. The structure was then embedded in the lipid bilayer consisting of DOPC and CLR in a 70:30 ratio using the packmol-memgen program in Ambertools 21^94^. Amber ff99SB-ILDN force field was used for protein parameters in GROMACS 2021^92^; ligand parameters and partial charges were assigned using the Open Force field^95^ and the AM1-BCC partial charge model, respectively. SLIPIDS parameters were used for lipids^96^. The simulation systems were solvated with the TIP3P^97^ water model. Counter ions were added to neutralize the net charge of the simulation systems. Hydrogen mass repartitioning scheme (HMR)^98,99^ was used to achieve a 4 fs integration timestep for all simulations; hydrogen masses, except those of water, were increased to 3 amu. Hydrogen motions were constrained using the LINCS algorithm^100,101^. In all cases, the simulation temperature was set to 298.15 K. A simulation pressure of 1 atmosphere was maintained using Berendsen barostat^102^ during equilibration with a time constant of 1 ps, followed by the Parrinello-Rahman barostat^103^ with a time constant of 2.0 ps for production simulations. A cut-off of 1 nm was used for short-range interactions, and long-range electrostatics were handled via PME^104,105^. The simulation system was minimized. Next, a five-step equilibration was used in which protein backbone atoms, non-hydrogen ligand atoms, and lipid head groups were restrained using the following force constants: 5, 2.5, 1, 0.5, and 0.1 kcal per mol per Å^2^. Then, an additional equilibration step was performed with position restrains only applied to the protein backbone and non-hydrogen ligand atoms of 0.1 kcal per mol per Å^2^. All simulations were conducted for 1 µs and in three replicates. The first 100 ns were treated as equilibration run, based on the area per lipid property. All analyses were performed based on the following 900 ns. The trajectory analysis was conducted using the GROMACS tools^92^ and the MDAnalysis package^93^. We investigated the structural stability with all-atom simulations where events can be observed in a timescale of about 500 ns. Thus, to observe relevant events, we conducted simulations for 1 µs. In addition, we performed martini simulations for 100 µs to investigate if CLR localizes around the protein. Both observations presented in this study are within reach of brute-force MD. The system composition of all simulation systems is summarized in Table 1 and the simulation equilibration status is shown in Supplementary Fig. S10.

**Table 1:** System composition of molecular dynamics simulations

	all-atom system 1	all-atom system 2	all-atom system 3	all-atom system 4	Martini system 1	Martini system 2
Protein	1	1	1	1	1	1
DOPC	144	144	143	146	153	153
Cholesterol	63	62	63	62	83	82
Ligand	1	1	0	0	0	0
Water	8021	8009	7998	8186	3529	3529
Cl ion	5	5	5	5	5	5

### Reporting summary

Further information on research design is available in the Nature Portfolio Reporting Summary linked to this article.

## Supplementary information

Supplementary Information

Reporting Summary

Transparent Peer Review file

## Source data

Source Data

## Data Availability

The cryo-EM maps of the full complexes (composite), the consensus maps, and the local refined maps have been deposited in the Electron Microscopy Data Bank (EMDB) with access numbers EMD-51288 (composite), EMD-51285 (consensus), EMD-51286 (focused map GPCR), and EMD-51287 (focused map G protein) for the GPR55-Gα_13_β_1_γ_2_-ScFv16-LPI complex and EMD-51284 (composite), EMD-51281 (consensus), EMD-51282 (focused map GPCR), and EMD-51283 (focused map G protein) for the GPR55-Gα_13_β_1_γ_2_-ScFv16-ML184. The structure coordinates of the GPR55-Gα_13_β_1_γ_2_-ScFv16-LPI and GPR55-Gα_13_β_1_γ_2_-ScFv16-ML184 complexes were deposited to the PDB with accession codes 9GE3 and 9GE2, respectively. The source data underlying Figs. 2b, d, f, 5b, c, and Supplementary Figs. S1e–f and S4 are provided as a Source Data file. Additional raw data that support the findings of this study are available from the corresponding author. The following PDB accession codes have been used as initial models (7YDH) or for structural comparison within this study: 2RH1^56^, 3SN6^57^, 7EW3^106^, 7T6B^42^, 7VIE^71^, 7TD0^70^, 7XZ5^68^, 7XV3^69^, 7YDH^82^, 8H8J^41^, 8SAI^67^. Molecular Dynamics simulations files and uncropped gels are available in the Source Data folder. Source data are provided in this paper.
