# Supplementary material for: Structural basis for lipid-mediated activation of G protein-coupled receptor GPR55
Source: Nat Commun. 2025 Feb 25;16:1973. doi: 10.1038/s41467-025-57204-y (PMC11861906; doi:10.1038/s41467-025-57204-y)
Supplement: Supplementary file 1 — Supplementary Information [file 41467_2025_57204_MOESM1_ESM.pdf]

Structural Basis for Lipid-mediated Activation of G Protein-coupled Receptor GPR55

Tobias Claff[1], Rebecca Ebenhoch[1], Jörg T. Kley[1], Aniket Magarkar[1], Herbert Nar[1], Dietmar Weichert[1]*

[1]Boehringer Ingelheim Pharma GmbH & Co. KG, Global Medicinal Chemistry, Birkendorfer Str. 65, 88397 Biberach an der Riß

**Corresponding author: dietmar.weichert@boehringer-ingelheim.com*

Table of contents

Supplementary Figures

Figure S1	Expression and purification of the GPR55-$G\alpha_{13}\beta_1\gamma_2$-ScFv16 complex with LPI and ML184
Figure S2	Cryo-EM data processing workflow
Figure S3	Cryo-EM Map of ligand binding pockets and ECL2 residues
Figure S4	Dose-response curves of G protein dissociation assays
Figure S5	Location of LPI and the palmitoyl-tail in the ligand binding pocket
Figure S6	N-linked glycosylation in the GPR55-ML184-structure with two initial GlcNAc units
Figure S7	Models of G152 single point mutations in the membrane opening
Figure S8	Comparison of conserved activation microswitches
Figure S9	Ligand recognition of selected different lipid receptors through their negatively charged moieties
Figure S10	Equilibration status of molecular dynamics (MD) simulations

Supplementary Tables

Table S1	List of GPCR structures determined in complex with Gα12 or Gα13 proteins as of May 2024.
Table S2	Cryo-EM data collection, refinement and validation statistics
Table S3	G protein dissociation assay results
Table S4	GPCR structures with resolved N- or O-linked glycosylation in the PDB as of December 2023.

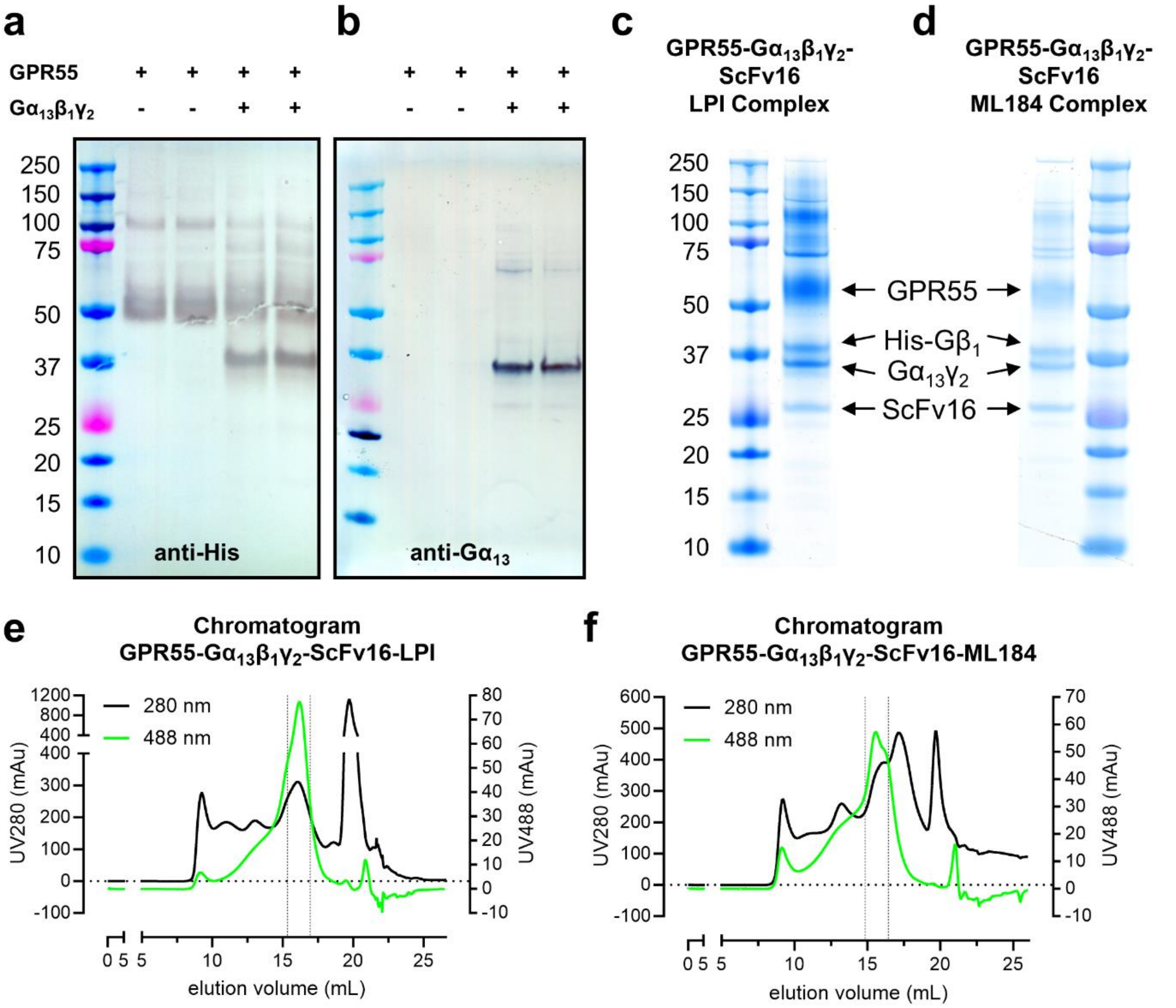

Supplementary Fig. S1. Expression and purification of the GPR55-G$\alpha_{13}\beta_1\gamma_2$-ScFv16 complex with LPI and ML184. a and **b** Western blot of Hi5 insect cell lysates expressing GPR55 alone or together with heterotrimeric G protein using **a** anti-His antibody or **b** anti-Gα_{13} antibody. **c** and **d** SDS-PAGE analysis of the final protein used for cryo-EM grid preparation for the **c** GPR55-G$\alpha_{13}\beta_1\gamma_2$-ScFv16-LPI complex or **d** GPR55-G$\alpha_{13}\beta_1\gamma_2$-ScFv16-ML184 complex. **e** and **f** Chromatogram of the **e** GPR55-G$\alpha_{13}\beta_1\gamma_2$-ScFv16-LPI complex or **f** GPR55-G$\alpha_{13}\beta_1\gamma_2$-ScFv16-ML184 during purification using a Superose™ 6 Increase 10/300 GL column on an ÄKTA pure™ chromatography system (Cytiva) equipped with a 10 mm flow cell. Peak fractions for each complex were pooled according to the GFP absorption peak at 488 nm as indicated by vertical dotted lines.

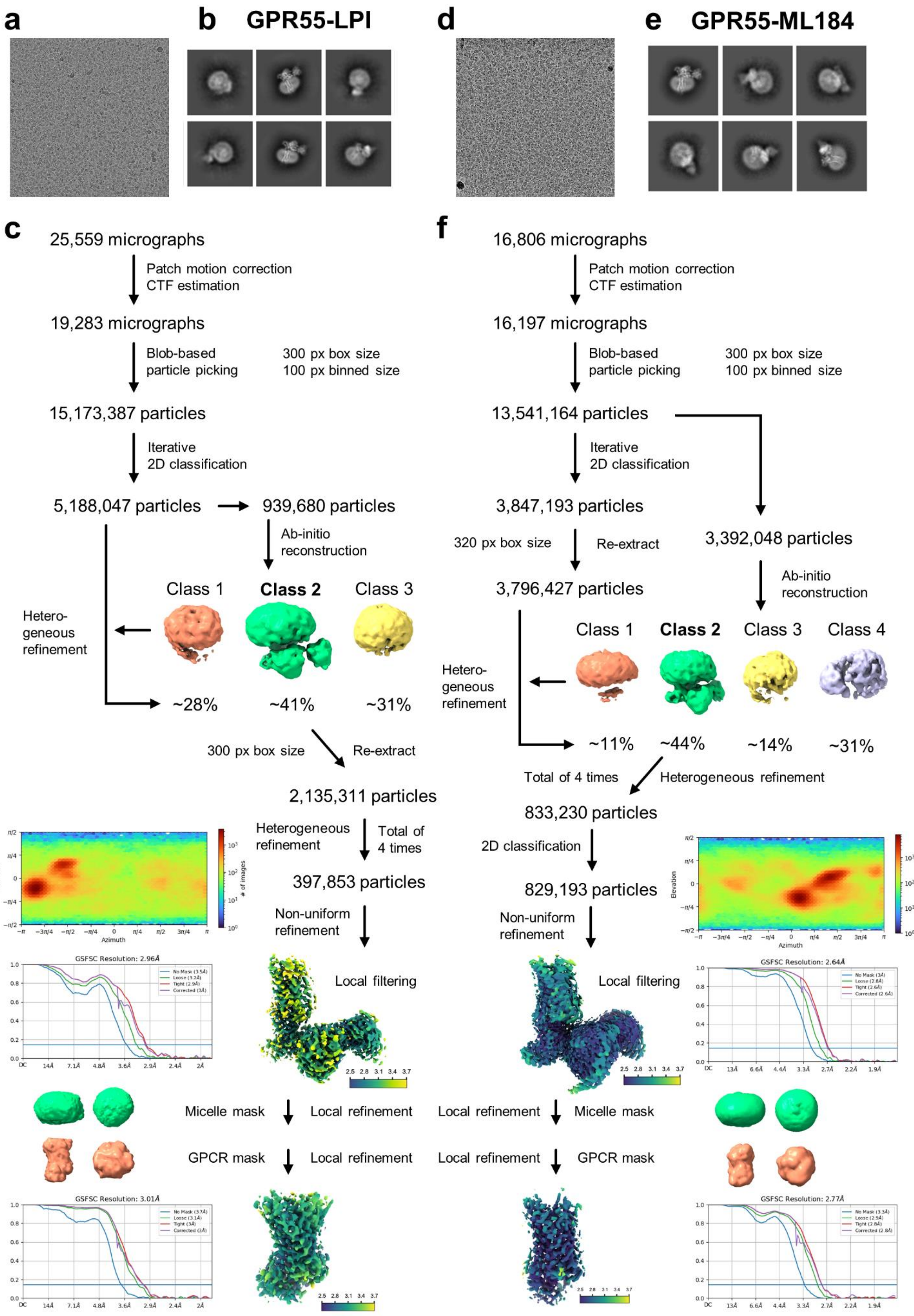

Supplementary Fig. S2. Cryo-EM data processing workflow.

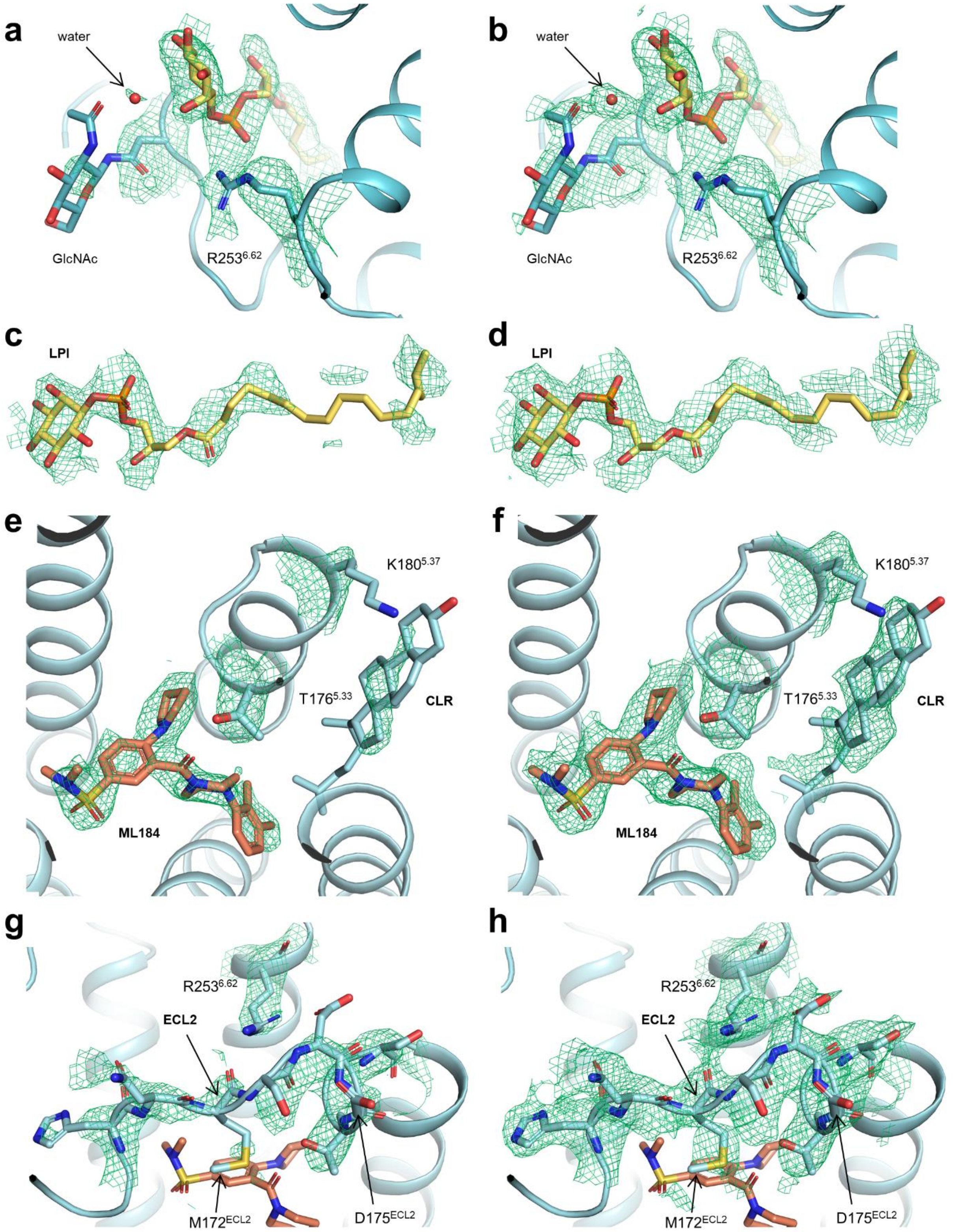

Supplementary Fig. S3. Cryo-EM Map of ligand binding pockets and ECL2 residues. a, b Cryo-EM map of the extracellular ends of LPI, GlcNAc, N171, and R253 at two different contour levels. **c, d** Cryo-EM map of LPI at two different contour levels. **e, f** Cryo-EM map of ML184, T176, CLR, and K180 at two different contour levels. **g, h** Cryo-EM map of ECL2 residues between S178 and H170 and R253 of the ML184 structure at two different contour levels.

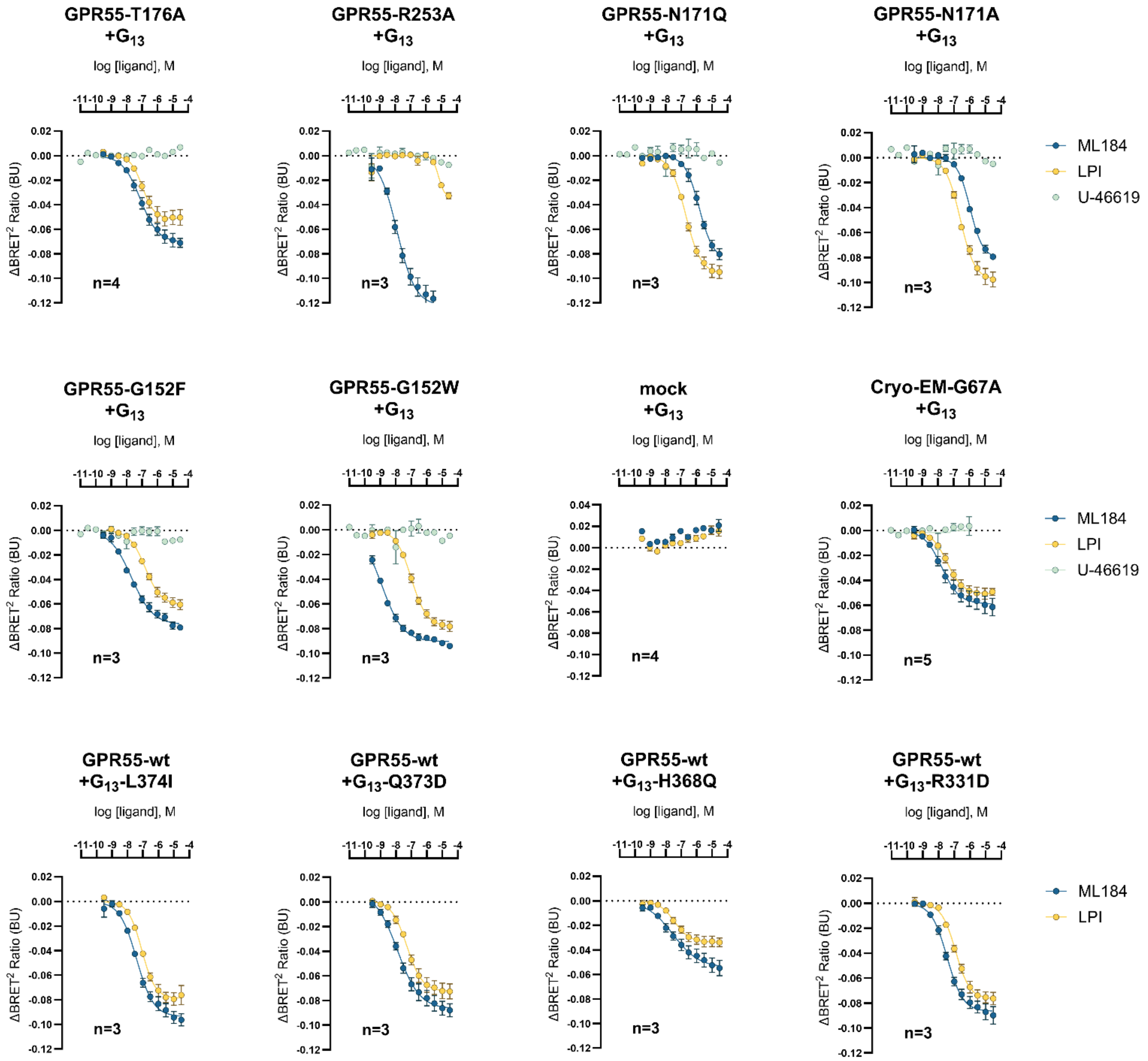

Supplementary Fig. S4. Dose-response curves of G protein dissociation assays. Data points represent means ± SEM from 3-7 independent experiments as indicated in Supplementary Table S3. Data of the negative control ligand U-46619 at GPR55 mutants represent means ± SEM from only two independent experiments.

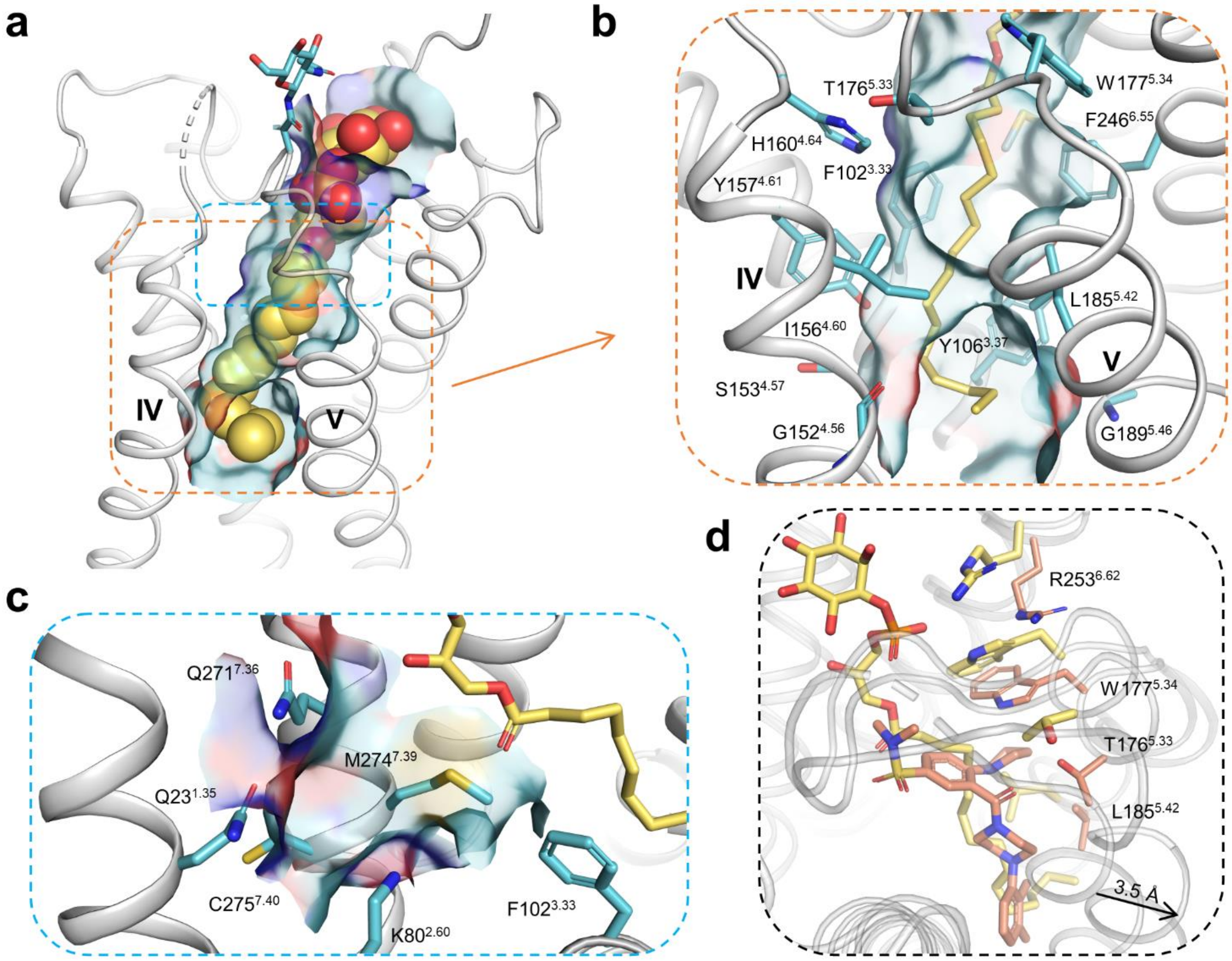

Supplementary Fig. S5. Location of LPI and the palmitoyl-tail in the ligand binding pocket. a Overview of the binding position of LPI (yellow spheres) in GPR55. **b** Magnified image of the binding pocket of the lipophilic palmitoyl-tail of LPI (yellow sticks) in the binding channel highlights the contacting amino acids. The binding pocket surface of LPI is shown in cyan in both panels. **c** Magnified image of the receptor cavity facing the glycerol hydroxy group. **d** Structural comparison of the binding pockets of LPI (yellow sticks) and ML184 (salmon sticks) shows rearrangements of several residues and an outward movement of the extracellular end of helix V to accommodate ML184.

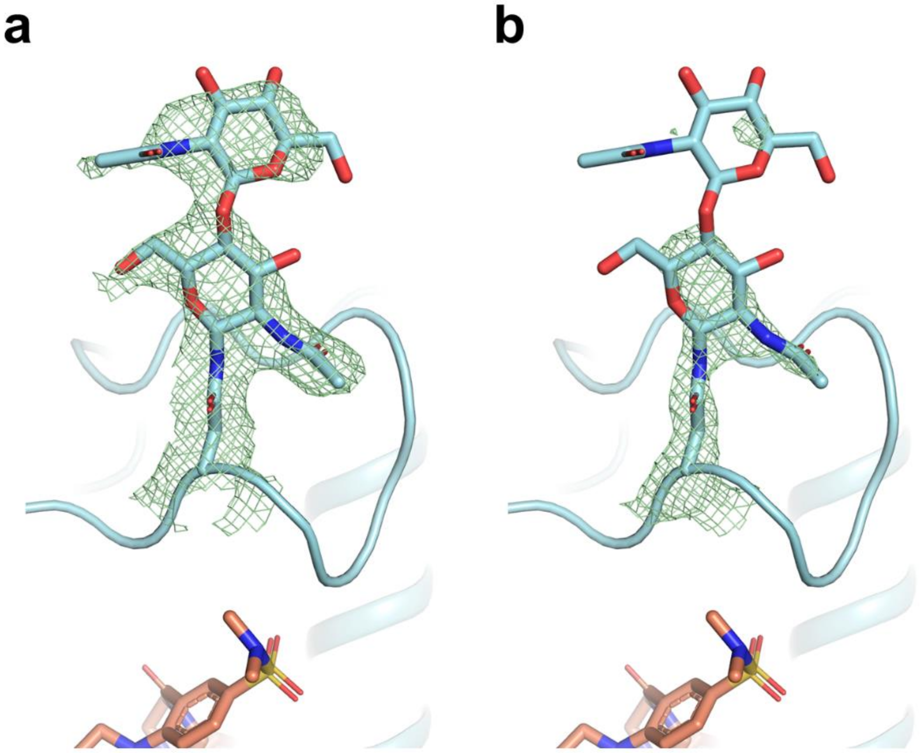

Supplementary Figure S6. N-linked glycosylation in the GPR55-ML184-structure with two initial GlcNAc units. a and **b** Cryo-EM map of N171^{ECL2} and the adjacent two glycan units are displayed with green mesh at different contour levels.

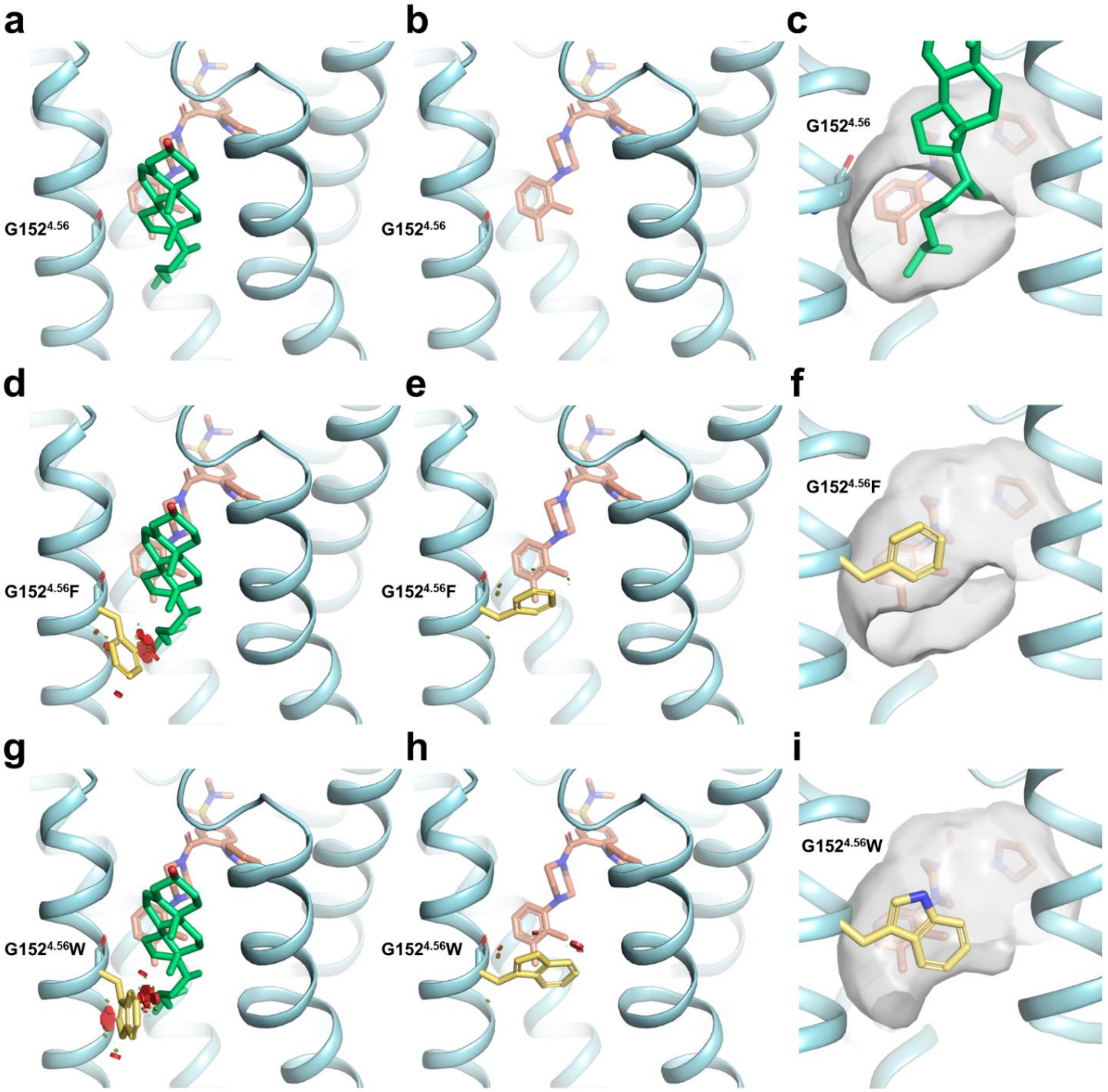

Supplementary Fig. S7. Models of G152 single point mutations in the membrane opening. The figure shows the membrane opening between helices IV and V **a, d, g** with CLR (green sticks) present and clashes displayed, **b, e, h** with CLR removed and clashes displayed, and **c, f, i** wt receptor or final models with ML184 (salmon sticks) surface displayed in gray. **a – c** shows the native receptor without mutation, **d – f** shows the receptor with G152$^{4.56}$F mutation, and **g – i** shows the receptor with G152$^{4.56}$W mutation (side chains shown as yellow sticks). Red disks indicate steric clashes. Point mutations of G152$^{4.56}$ to either F or W were introduced using PyMOL.

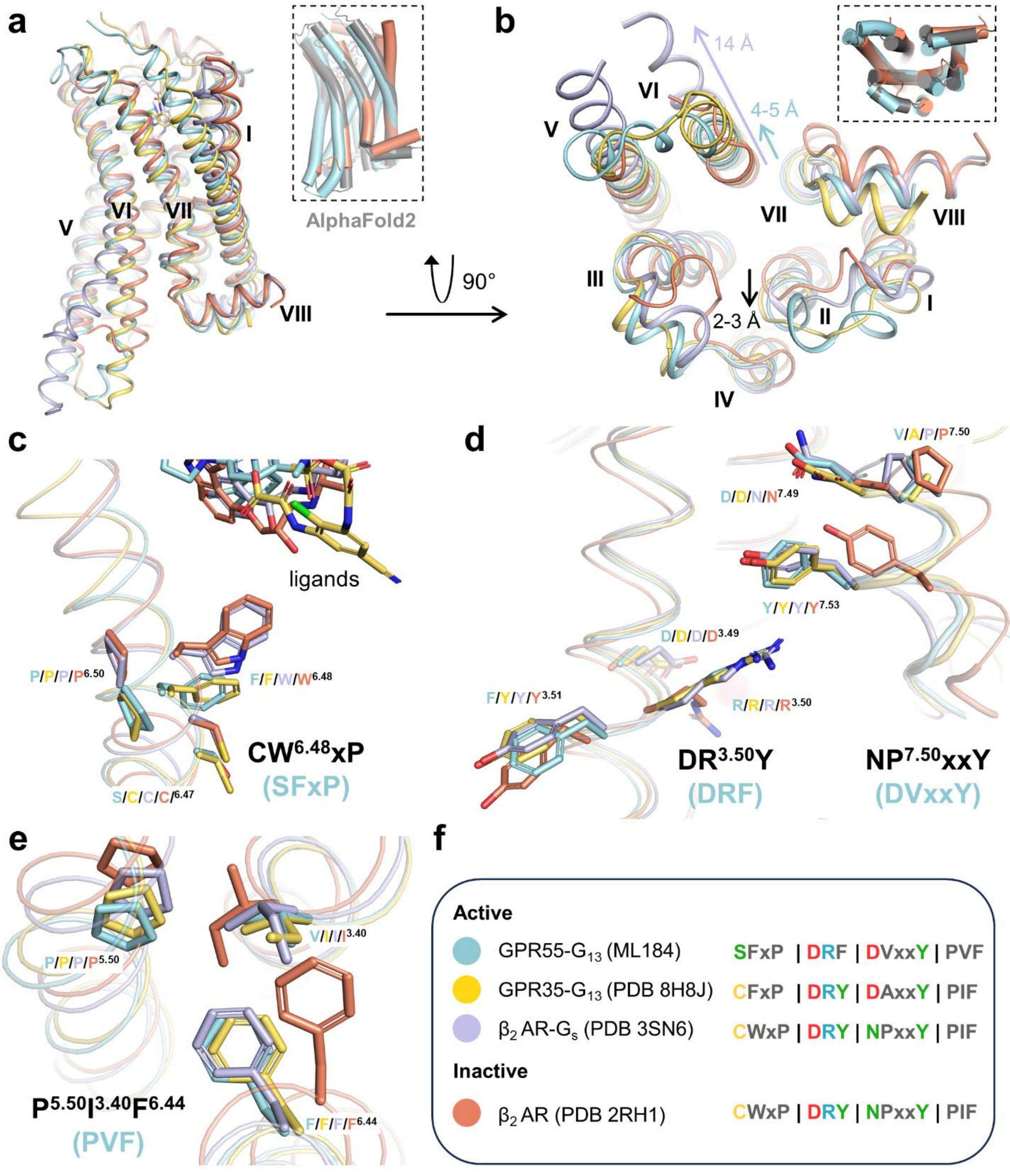

Supplementary Fig. S8. Comparison of conserved activation microswitches. Superimposition of GPR55-G_{13} (active structure, blue), GPR35-G_{13} (active structures, yellow, PDB 8H8J), β_2 adrenergic receptor (active structure, violet, PDB 3SN6), β_2AR (inactive structure, orange, PDB 2RH1). An inactive state AlphaFold model from GPCRdb (gray) is superimposed separately (rectangle box) with GPR55 and the inactive β_2AR using a cylindrical helix representation. **a** Alignment focused on the transmembrane helices. **b** Intracellular view of the helix bundle highlights minor outward helix IV outward movement of GPR55 in comparison to the inactive state structure. Different activation microswitches were compared focused on **c** CWxP motif, **d** DRY and NPxxY motif, and **e** PIF motif. **f** Color coding and sequence alignment of compared microswitches between all four receptors.

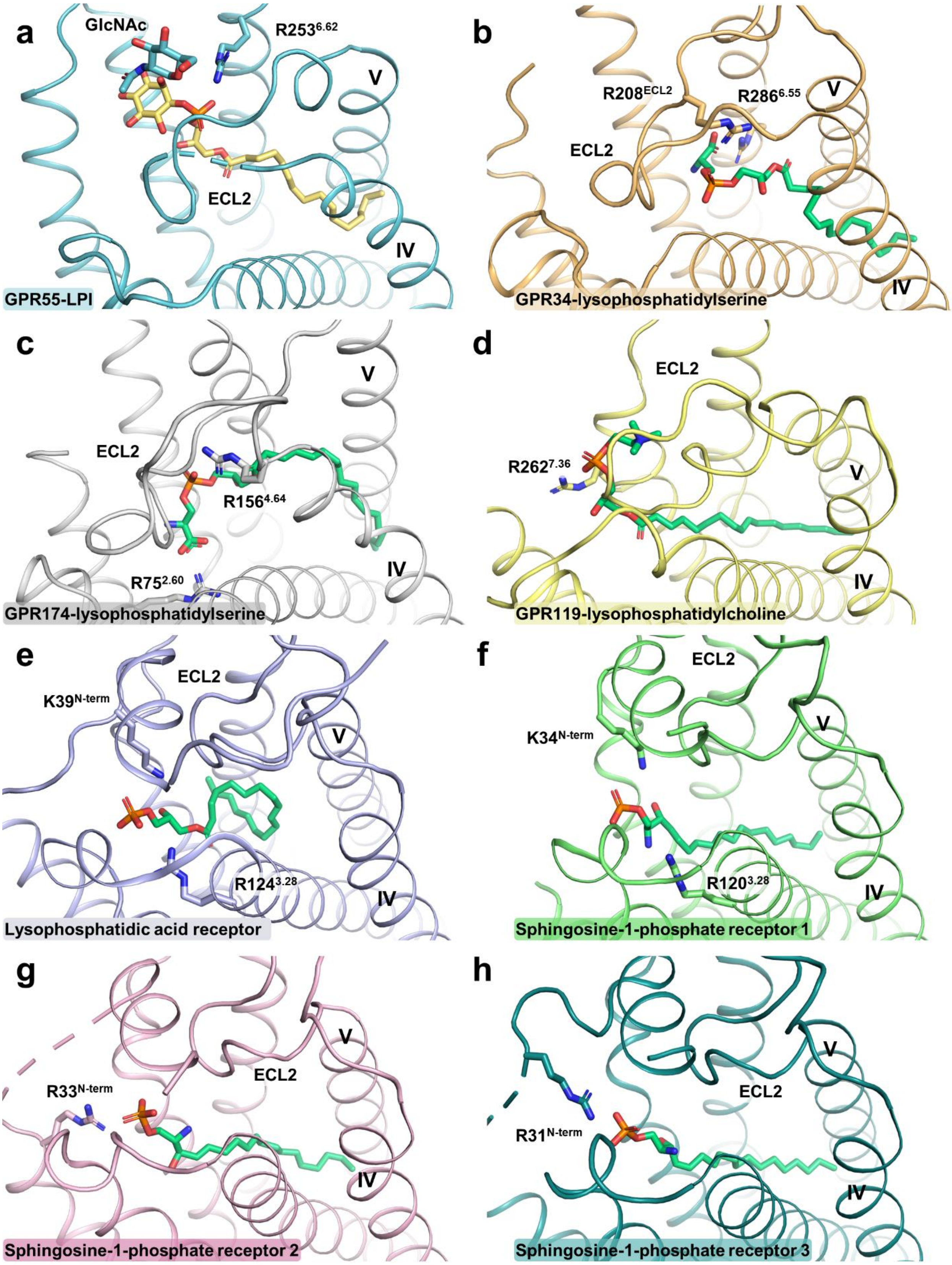

Supplementary Fig. S9. Ligand recognition of selected different lipid receptors through their negatively charged moieties. GPCR-lipid interactions of **a** GPR55-LPI (this study), **b** GPR34-LPS (PDB 8SAI), **c** GPR174-LPS (PDB 7XV3), **d** GPR119-LPC (PDB 7XZ5), **e** $LPAR_1$-LPA (PDB 7TD0), **f – h** and $S1P_{1\text{-}3}$-S1P (PDB 7VIE, 7T6B, and 7EW3). The same perspective was used for all binding pockets.

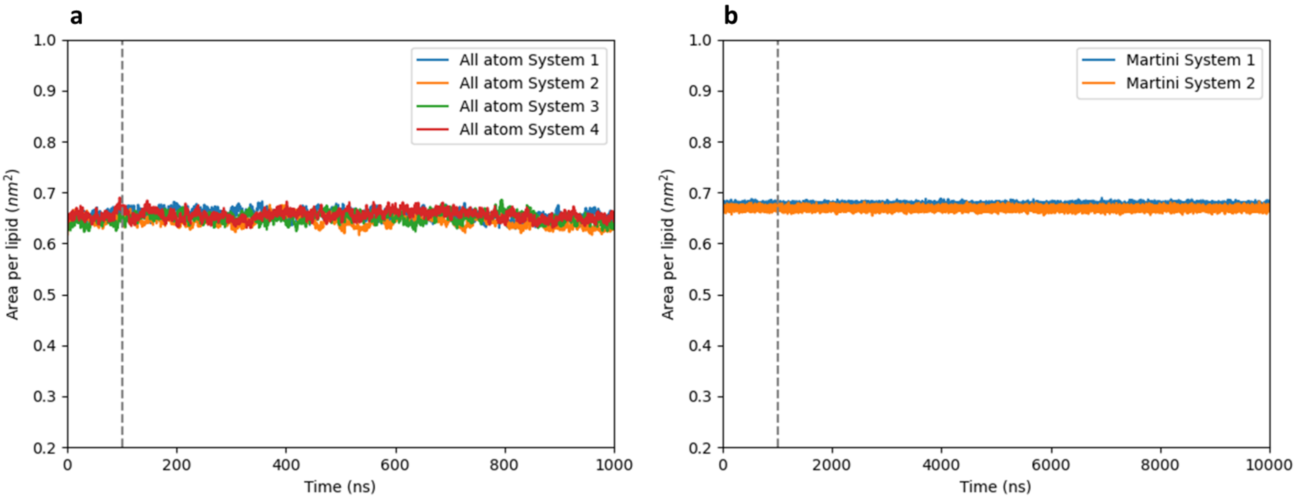

Supplementary Fig. S10. Equilibration status of molecular dynamics (MD) simulations. The equilibration of the MD systems is illustrated by a plot showing the area per lipid over time for the all-atom **a**, and martini simulations **b**. The naming conventions for the systems align with those detailed in the Methods section. The area per lipid is calculated as simulation box dimension x, multiplied by simulation box dimension y and divided by the number of DOPC and Cholesterol molecules in a single leaflet of lipid bilayer. **a** The all-atom simulations were conducted over a period of 1 microsecond (µs), with the initial 100 nanoseconds (ns) serving as the equilibration phase. The subsequent 900 ns were used for analysis. A dotted vertical line marks the transition from equilibration to analysis. **b** In case of the martini simulations, the first 1 µs was dedicated to equilibration, followed by a 9 µs period for analysis. The transition from equilibration to analysis is indicated by a dotted vertical line.

Supplementary Table S1. List of GPCR structures determined in complex with Gα$_{12}$ or Gα$_{13}$ proteins as of July 2024.

Receptor	Abbreviation	Family	G protein subtype	Ligand	PDB ID	FSC Resolution
Adhesion G protein-coupled receptor L3	AGRL3	Adhesion	Gα$_{13}$	apo	7SF7	2.90 Å
Adhesion G protein-coupled receptor G1	AGRG1	Adhesion	Gα$_{13}$	apo	7SF8	2.70 Å
Sphingosine-1-phosphate receptor 2	S1P$_2$R	Rhodopsin	Gα$_{13}$	S1P	7T6B	3.19 Å
Adhesion G protein-coupled receptor F1	AGRF1	Adhesion	Gα$_{13}$	apo	7WY0	2.83 Å
Adhesion G protein-coupled receptor F1	AGRF1	Adhesion	Gα$_{12}$	apo	7WZ7	2.83 Å
Adhesion G protein-coupled receptor L3	AGRL3	Adhesion	Gα$_{12}$	apo	7X10	2.93 Å
Adhesion G protein-coupled receptor E5	AGRE5	Adhesion	Gα$_{13}$	apo	7YDH	3.10 Å
Adhesion G protein-coupled receptor E5	AGRE5	Adhesion	Gα$_{12}$	apo	7YDJ	3.03 Å
GPR35	GPR35	Rhodopsin	Gα$_{13}$	Iodox-amide	8H8J	3.20 Å
GPR55 (this publication)	**GPR55**	**Rhodopsin**	**Gα$_{13}$**	**LPI**	**9GE3**	**2.96 Å**
GPR55 (this publication)	**GPR55**	**Rhodopsin**	**Gα$_{13}$**	**ML184**	**9GE2**	**2.64 Å**

Supplementary Table S2. Cryo-EM data collection, refinement and validation statistics.

	**GPR55-Gα$_{13}$β$_1$γ$_2$-ScFv16-LPI** (PDB 9GE3)	**GPR55-Gα$_{13}$β$_1$γ$_2$-ScFv16-ML184** (PDB 9GE2)
**Data collection and processing**		
Magnification	130,000	105,000
Voltage (kV)	300	300
Detector	Falcon IV	Gatan K3
Electron exposure (e^-/Å^2)	40	45
Defocus range (μm)	-2.54 to -0.40	-3.56 to -0.88
Pixel size (Å)	0.951	0.829
Symmetry imposed	C1	C1
Initial particle images (no.)	5,188,047	13,541,164
Final particle images (no.)	397,853	829,193
Map resolution (Å)*<br>FSC threshold 0.143	2.87 (composite map)<br>2.96 (consensus map)<br>3.01 (focused map GPCR)<br>2.88 (focused map G protein)	2.51 (composite map)<br>2.64 (consensus map)<br>2.77 (focused map GPCR)<br>2.51 (focused map G protein)
**Refinement**		
Initial model used (PDB code)	ML184 structure	AlphaFold (GPR55) & 7YDH
Map sharpening *B* factor (Å^2)	109.2 (consensus map)<br>115.6 (focused map GPCR)<br>96.4 (focused map G protein)	98.6 (consensus map)<br>102.5 (focused map GPCR)<br>87.5 (focused map G protein)
Model composition		
Non-hydrogen atoms	8,563	9,082
Protein residues	1,078	1,137
Ligands (lipids)	1 (0)	1 (1)
Waters	6	15
B factors (Å^2)		
Protein	49.3	37.5
Ligand	29.6	17.2
RMSD		
Bond lengths (Å)	0.002	0.003
Bond angles (°)	0.474	0.535
Validation		
MolProbity score	1.95	1.70
Clashscore	8.86	5.11
Poor rotamers (%)	3.07	2.82
Ramachandran plot		
Favored (%)	97.46	97.60
Allowed (%)	2.54	2.40
Disallowed (%)	0	0

Supplementary Table S3. G protein dissociation assay results.*

Receptor/ Mutant	G protein	Basal activation ± SEM (%)	Ligand	pEC_{50} ± SEM	Adjusted P value vs. wt†	Efficacy ± SEM (%)	No. of independent experiments
**wt GPR55**	$G\alpha_{13}$	66 ± 2	ML184	7.35 ± 0.07	n.a.	100 ± 2	7
			LPI	7.45 ± 0.08	n.a.	86 ± 2	7
**wt GPR55**	$G\alpha_{12}$	13 ± 5	ML184	n.d.	n.a.	n.d.	4
			LPI	n.d.	n.a.	n.d.	4
**Cryo-EM construct**	$G\alpha_{13}$	49 ± 6	ML184	7.74 ± 0.03	0.0010	86 ± 2	5
			LPI	7.40 ± 0.06	0.9968	82 ± 4	5
**T176A**	$G\alpha_{13}$	50 ± 1	ML184	7.11 ± 0.08	0.0377	100 ± 1	4
			LPI	6.96 ± 0.02	0.0001	87 ± 2	4
**R253A**	$G\alpha_{13}$	13 ± 3	ML184	7.84 ± 0.08	0.0005	103 ± 3	3
			LPI	5.17 ± 0.09	<0.0001	37 ± 2	3
**N171Q**	$G\alpha_{13}$	19 ± 2	ML184	5.85 ± 0.07	<0.0001	76 ± 2	3
			LPI	6.72 ± 0.08	<0.0001	85 ± 3	3
**N171A**	$G\alpha_{13}$	16 ± 3	ML184	5.98 ± 0.04	<0.0001	73 ± 3	3
			LPI	6.61 ± 0.06	<0.0001	85 ± 3	3
**G152F**	$G\alpha_{13}$	52 ± 3	ML184	7.66 ± 0.06	0.0375	108 ± 3	3
			LPI	6.79 ± 0.02	<0.0001	94 ± 3	3
**G152W**	$G\alpha_{13}$	41 ± 2	ML184	8.87 ± 0.01‡	<0.0001	107 ± 1	3
			LPI	7.00 ± 0.03	0.0009	96 ± 5	3
**TBXA2**	$G\alpha_{13}$	-15 ± 14	U-46619	9.03 ± 0.11	n.a.	100 ± 2	3
	$G\alpha_{12}$	-6 ± 15	U-46619	7.62 ± 0.15	n.a.	100 ± 8	3
**wt GPR55**	$G\alpha_{13}$-L374I	17 ± 3	ML184	7.44 ± 0.05	0.8504	84 ± 1	3
			LPI	7.09 ± 0.07	0.0121	71 ± 3	3
	$G\alpha_{13}$-Q373D	33 ± 11	ML184	7.77 ± 0.04	0.0058	94 ± 7	3
			LPI	7.28 ± 0.01	0.3500	84 ± 8	3
	$G\alpha_{13}$-H368Q	64 ± 8	ML184	7.53 ± 0.09	0.3466	101 ± 4	3
			LPI	7.45 ± 0.03	>0.9999	88 ± 6	3
	$G\alpha_{13}$-R331D	27 ± 7	ML184	7.47 ± 0.10	0.6846	89 ± 3	3
			LPI	6.92 ± 0.01	0.0005	80 ± 4	3

*The degree of activation in % was calculated by normalization of $BRET^2$ ratios ($BRET^2$ ratio of 30 μM ligand for efficacy or basal $BRET^2$ ratios of 1.5% DMSO) to the respective $BRET^2$ ratios for the wt GPR55 plus G_{13}-biosensor at 30 μM ML184 (100% activation) and for a mock-transfection plus G_{13}-biosensor (0% activation). The same normalization approach was used for the $G\alpha_{13}$ mutants.

[†]P values were calculated by one-way ANOVA with Dunnett's post-hoc test for all mutants versus wt GPR55 separately for each ligand (seven comparisons each). The ANOVA P values equal <0.0001 for both ligand comparisons. The $G\alpha_{13}$ mutants were tested independently versus wt GPR55 plus wt $G\alpha_{13}$. Here, the ANOVA P value equals 0.0237 for the ML184 comparison and 0.0008 for the LPI comparison, respectively.

[‡]The pEC_{50} value of ML184 at GPR55 mutant G152W was calculated by extrapolating $\Delta BRET^2$ ratios for concentrations <0.3 nM. n.d.: not determined. n.a. not applicable

Supplementary Table S4. GPCR structures with resolved N- or O-linked glycosylation in the PDB as of July 2024.

PDB ID	Receptor	Ligand distance within 5 Å	Residue(s)	Location	Glycan units
**Class A GPCRs**					
**9GE2, 9GE3 (this publication)**	**GPR55**	**Yes**	**N171**	**ECL2**	**1-2**
2J4Y, 3C9M, 3PQR, 6FKB, 6FUF, 7ZBC, 7ZBE	Rhodopsin	no	N2<br>N15	*N*-term	0-1<br>1-4
3V2W, 3V2Y, 7EVY, 7TD3, 7TD4	$S1PR_1$	no	N30	*N*-term	1
7YXA	$S1PR_5$	no	N20	*N*-term	1
7SRQ, 7SRS	Serotonin $5\text{-}HT_{2B}$ receptor	no	N204	ECL2	1
6DO1, 6OS2	Angiotensin II receptor type 1	no	N176	ECL2	1
6MEO	Chemokine receptor type 5	no	S7	*N*-term	1
7RKF	US28	no	N9	*N*-term	1
**Class B GPCRs**					
5NX2, 6LN2	Glucagon-like peptide 1	no	N63<br>N82	*N*-term	0-1<br>2
5XEZ, 5XF1	Glucagon receptor	no	N46<br>N59<br>N74<br>N78	*N*-term	1<br>2-3<br>2<br>2
7TYF, 7TYI, 7TYN, 7TYO, 7TYX, 7TYW, 7TZF, 8F0J, 8F0K, 8F2A, 8F2B, 9AUC	Calcitonin and Amylin 1-3 receptors	no	N73<br>N125<br>N130	*N*-term	0-1<br>0-1<br>1
**Class C GPCRs**					
6N51, 6N52, 7FD8, 7FD9	Metabotropic glutamate receptor 5	no	N210<br>N445	*N*-term	1<br>0-1
**Class F GPCRs**					
5L7D, 5L7I, 5V56, 7ZI0	Smoothened receptor	no	N188<br>N493	*N*-term<br>6.74	0-1<br>0-1
