# Supplementary material for: Structural basis for lipid-mediated activation of G protein-coupled receptor GPR55
Source: Nat Commun. 2025 Feb 25;16:1973. doi: 10.1038/s41467-025-57204-y (PMC11861906; doi:10.1038/s41467-025-57204-y)
Supplement: Supplementary file 2 — Reporting Summary [file 41467_2025_57204_MOESM2_ESM.pdf]

nature portfolio

Corresponding author(s): Dr. Dietmar Weichert

Last updated by author(s): Dec 23, 2024

Reporting Summary

Nature Portfolio wishes to improve the reproducibility of the work that we publish. This form provides structure for consistency and transparency in reporting. For further information on Nature Portfolio policies, see our Editorial Policies and the Editorial Policy Checklist.

Statistics

For all statistical analyses, confirm that the following items are present in the figure legend, table legend, main text, or Methods section.

n/a	Confirmed	
☐	☒	The exact sample size (n) for each experimental group/condition, given as a discrete number and unit of measurement
☐	☒	A statement on whether measurements were taken from distinct samples or whether the same sample was measured repeatedly
☐	☒	The statistical test(s) used AND whether they are one- or two-sided<br>*Only common tests should be described solely by name; describe more complex techniques in the Methods section.*
☒	☐	A description of all covariates tested
☒	☐	A description of any assumptions or corrections, such as tests of normality and adjustment for multiple comparisons
☐	☒	A full description of the statistical parameters including central tendency (e.g. means) or other basic estimates (e.g. regression coefficient) AND variation (e.g. standard deviation) or associated estimates of uncertainty (e.g. confidence intervals)
☐	☒	For null hypothesis testing, the test statistic (e.g. F, t, r) with confidence intervals, effect sizes, degrees of freedom and P value noted<br>*Give P values as exact values whenever suitable.*
☒	☐	For Bayesian analysis, information on the choice of priors and Markov chain Monte Carlo settings
☒	☐	For hierarchical and complex designs, identification of the appropriate level for tests and full reporting of outcomes
☒	☐	Estimates of effect sizes (e.g. Cohen's d, Pearson's r), indicating how they were calculated

Our web collection on statistics for biologists contains articles on many of the points above.

Software and code

Policy information about availability of computer code

Data collection	Cryo-EM data collection using a Titan Krios G4 microscope and the Thermo Scientific software (EPU), BMG Labtech PHERAstar FSX
Data analysis	CryoSPARC v4.4, UCSF ChimeraX v1.6.1, UCSF Chimera v1.16, Coot v0.9.8.95, PyMOL v2.5.7, Phenix v1.21.1_5286, GraphPad Prism v10, AlphaFold v2

For manuscripts utilizing custom algorithms or software that are central to the research but not yet described in published literature, software must be made available to editors and reviewers. We strongly encourage code deposition in a community repository (e.g. GitHub). See the Nature Portfolio guidelines for submitting code & software for further information.

Data

Policy information about availability of data

All manuscripts must include a data availability statement. This statement should provide the following information, where applicable:

- Accession codes, unique identifiers, or web links for publicly available datasets
- A description of any restrictions on data availability
- For clinical datasets or third party data, please ensure that the statement adheres to our policy

The cryo-EM maps of the full complexes (composite), the consensus maps, and the local refined maps have been deposited in the Electron Microscopy Data Bank (EMDB) with access numbers EMD-51288 (composite), EMD-51285 (consensus), EMD-51286 (focused map GPCR), and EMD-51287 (focused map G protein) for the GPR55-Gα13β1γ2-ScFv16-LPI complex and EMD-51284 (composite), EMD-51281 (consensus), EMD-51282 (focused map GPCR), and EMD-51283 (focused map G

protein) for the GPR55-Gα13β1γ2-ScFv16-ML184. The structure coordinates of the GPR55-Gα13β1γ2-ScFv16-LPI and GPR55-Gα13β1γ2-ScFv16-ML184 complexes were deposited to the PDB with accession codes 9GE3 and 9GE2, respectively. Upon reasonable request, additional raw data that support the findings of this study are available from the corresponding author.

Human research participants

Policy information about studies involving human research participants and Sex and Gender in Research.

Reporting on sex and gender	not applicable
Population characteristics	not applicable
Recruitment	not applicable
Ethics oversight	not applicable

Note that full information on the approval of the study protocol must also be provided in the manuscript.

Field-specific reporting

Please select the one below that is the best fit for your research. If you are not sure, read the appropriate sections before making your selection.

☒ Life sciences ☐ Behavioural & social sciences ☐ Ecological, evolutionary & environmental sciences

For a reference copy of the document with all sections, see nature.com/documents/nr-reporting-summary-flat.pdf

Life sciences study design

All studies must disclose on these points even when the disclosure is negative.

Sample size	Statistical methods to predetermine sample sizes were not applied. The total number of particles for structure determination resulted from automatic picking using cryo-EM micrographs. Functional assays were performed in three independent experiments.
Data exclusions	No data was excluded from the analyses.
Replication	Functional data was collected in at least three independent experiments.
Randomization	Randomization was not relevant to the study and cryo-EM data was collected automatically.
Blinding	Blinding was not relevant to the study.

Reporting for specific materials, systems and methods

We require information from authors about some types of materials, experimental systems and methods used in many studies. Here, indicate whether each material, system or method listed is relevant to your study. If you are not sure if a list item applies to your research, read the appropriate section before selecting a response.

Materials & experimental systems

n/a	Involved in the study	
☐	☒	Antibodies
☐	☒	Eukaryotic cell lines
☒	☐	Palaeontology and archaeology
☒	☐	Animals and other organisms
☒	☐	Clinical data
☒	☐	Dual use research of concern

Methods

n/a	Involved in the study	
☒	☐	ChIP-seq
☒	☐	Flow cytometry
☒	☐	MRI-based neuroimaging

Antibodies

Antibodies used	anti-penta-His (Thermo Fisher Scientific, cat. #P-21315), anti-Gα13 (ABclonal, cat. #A20908), anti-mouse-igG (Sigma, cat. #A4312), and anti-rabbit igG (Sigma, cat. #A3687)
Validation	All antibodies are commercially available and were validated by the manufacturers:

Validation | anti-penta-His: https://www.thermofisher.com/antibody/product/Penta-His-Tag-Antibody-Monoclonal/P-21315
anti-Gα13: https://eu.abclonal.com/catalog-antibodies/GNA13RabbitmAb/A20908
anti-mouse: https://www.sigmaaldrich.com/DE/en/product/sigma/a4312
anti-rabbit: https://www.sigmaaldrich.com/DE/en/product/sigma/a3687

Eukaryotic cell lines

Policy information about cell lines and Sex and Gender in Research

Cell line source(s): Mammalian cell line HEK293H (ThermoFisher, cat. #11631017), Insect cell lines HighFive and Sf9 (ThermoFisher, cat. #11496015)

Authentication: Independent cell line authentication was not performed.

Mycoplasma contamination: The above cell lines were not tested for Mycoplasma contamination after purchase.

Commonly misidentified lines (See ICLAC register): No commonly misidentified cell lines were used.
