# Supplementary material for: Structural basis for lipid-mediated activation of G protein-coupled receptor GPR55
Source: Nat Commun. 2025 Feb 25;16:1973. doi: 10.1038/s41467-025-57204-y (PMC11861906; doi:10.1038/s41467-025-57204-y)
Supplement: Supplementary file 4 — Source Data [file 41467_2025_57204_MOESM4_ESM.zip › Source Data/uncropped gels.pdf]

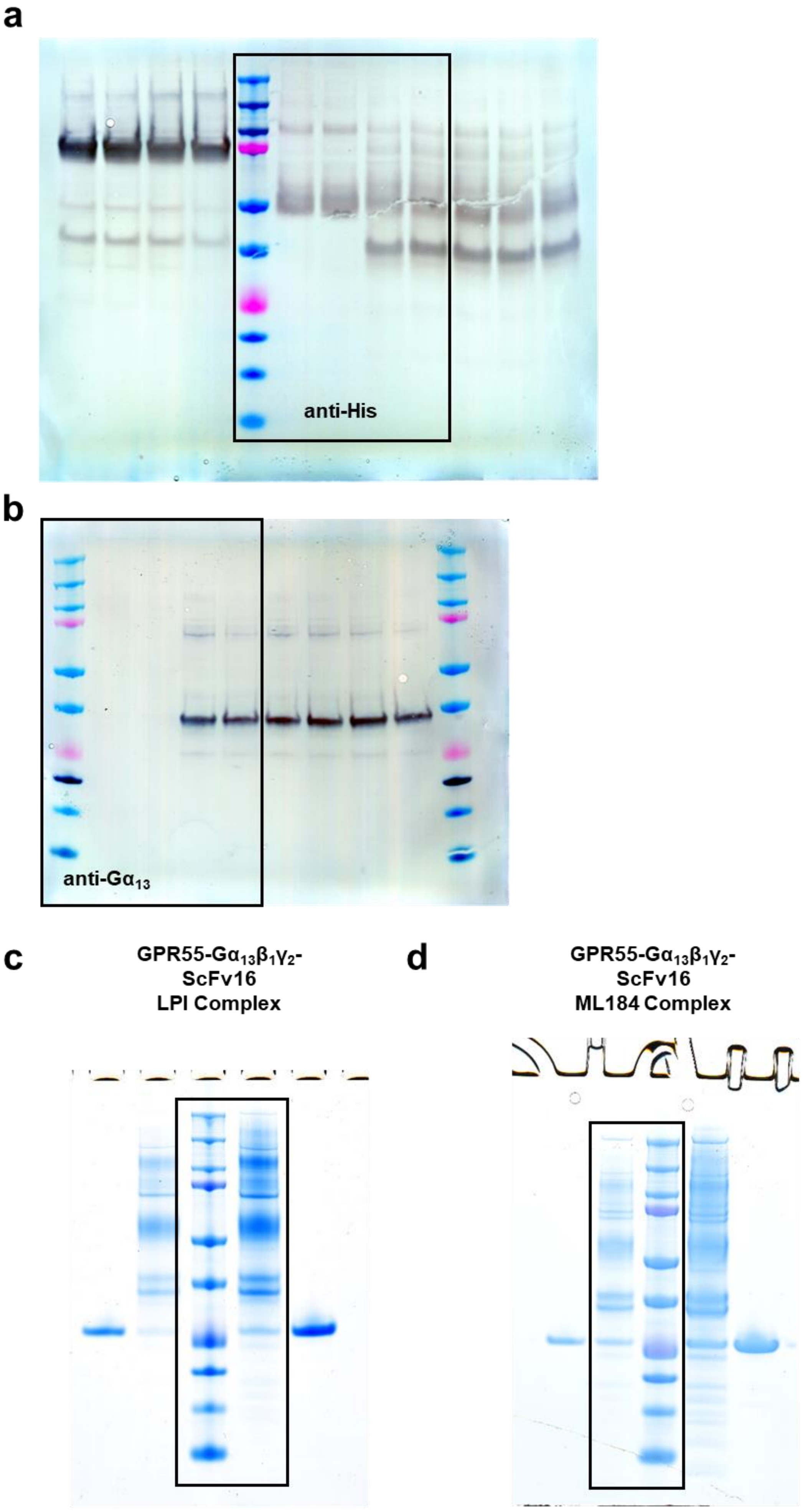
a
anti-His
b
anti-Gα13
c
GPR55-Gα13β1γ2-
ScFv16
LPI Complex
d
GPR55-Gα13β1γ2-
ScFv16
ML184 Complex
