# Supplementary material for: Structural basis for lipid-mediated activation of G protein-coupled receptor GPR55
Source: Nat Commun. 2025 Feb 25;16:1973. doi: 10.1038/s41467-025-57204-y (PMC11861906; doi:10.1038/s41467-025-57204-y)
Supplement: Supplementary file 3 — Transparent Peer Review file [file 41467_2025_57204_MOESM3_ESM.pdf]

nature portfolio

Peer Review File

Structural Basis for Lipid-mediated Activation of G Protein-coupled Receptor GPR55

Corresponding Author: Dr Dietmar Weichert

This file contains all reviewer reports in order by version, followed by all author rebuttals in order by version.

Attachments originally included by the reviewers as part of their assessment can be found at the end of this file.

Version 0:

Reviewer comments:

Reviewer #1

(Remarks to the Author)
In their study, Tobias Claff et al. investigated the structures of the orphan lipid receptor GPR55, an emerging drug target, in complex with a putative endogenous agonist, 1-palmitoyl-2-lysophosphatidylinositol (LPI), and a synthetic agonist, ML184. The authors expand their structural findings with data from G protein recruitment experiments, incorporating various point mutations and molecular dynamics simulations to validate the structures and further explore the interaction interfaces. Notably, the ligand density in the LPI-bound structure suggests a direct interaction between the ligand and an N-linked glycan. Furthermore, in both structures, the ligands face the lipid bilayer, with a cholesterol-like binder attached to the receptor that interacts with the ligands. The structures of GPR55 have not been published before, making these insights novel and certainly deserving of publication in Nature Communications.
The paper is well-written, the figures are clear, and the densities obtained by cryo-EM are of high quality, accurately reflecting the experimental data. I did not identify any seriously misleading elements in the manuscript; however, I strongly recommend addressing several points before publication.
Major Points:
The contact between the modelled cholesterol and ligands is discussed repeatedly in the manuscript. However, cholesterol hemisuccinate tris (CHS-Tris) was present during solubilization and in all purification buffers, including the final size-exclusion buffer. It is plausible that a CHS molecule could bind instead of cholesterol, given these conditions. The structural difference between cholesterol and CHS lies in a distant region of the molecule that may not be fully resolved. Please clarify why cholesterol is present unambiguously or rephrase where necessary.
While G protein dissociation data is valuable, it should be interpreted with caution. Differences between mutants may be influenced by variations in expression levels. Especially, when glycosylation is affected (which might alter receptor folding and trafficking). This concern does not apply to EC50 values, but efficacy and basal activity values may have been affected. Was there a control for mutant expression/surface expression levels? If not, please, state it in the text and make it explicit that the data might have been affected. If yes, please, provide the data.
Although not a major issue, there is a sense that with such a high starting number of micrographs and particles, the resolution of the structures could be pushed even further. It appears that the authors have not attempted to correct for motion per particle (e.g., Bayesian polishing in Relion or Reference-Based Motion Correction in recent CryoSPARC versions). Additionally, CTF refinement, per-particle defocus refinement, and splitting into AFIS groups>separately correcting for the beam tilt, etc. might improve the resolution. It is not uncommon to achieve a resolution improvement of 0.1-0.3 Å using some or all of these techniques.

Minor points:

I suggest adding the tissue expression pattern of GPR55 to the introduction for context.
How similar are the two solved structures? What is the root mean square deviation (r.m.s.d.) between them?
To be precise, I recommend replacing “endogenous ligand” (referring to LPI) with “putative endogenous ligand.”
In the section discussing GPR55 activation, only the higher-resolution structure is considered. There is no mention of the activation microswitches comparison between the two solved structures. Are they the same? This information would be

interesting to include.
I recommend revisiting the modelled water molecules to take a more conservative approach, considering densities on the focused map and the local resolution of the region. At least two of them, HOH5 and HOH6 (as shown in the attached figures), appear to have very weak densities compared to the others and the neighbouring side chains.
While not essential, it would be beneficial to include a comparison with the positions of canonical activation switches (such as in the β2 adrenergic receptor) if claiming that the activation pathway differs from the canonical one.
Another example of a lipid receptor with a suggested opening between helices IV and V is the CysLTR1 receptor. As far as I recall, there were even molecular dynamics simulations of the lateral ligand entrance (Luginina et al. 2019).

Figures:
Main Figure 1 (a and e): The transparent density at a different contour level (likely due to the micelle visualization) is somewhat confusing, particularly the top blobs that originally belonged to the protein. I encourage the authors to explore options in Chimera to mitigate this effect.
Main Figure 2 (a): Please add a label for residue 171, as described in the text.
Main Figure 5 (e): Please specify which map is depicted.

Line by line:
Line 10: It appears that “activation of lipid GPCRs” or something similar is missing. The sentence does not seem complete.
Line 16: I suggest removing “(endogenous)” for clarity.
Line 39: A reference to the reported selective G13 coupling of GPR55 is missing.
Line 357: While you mention this in the discussion, it would be helpful to note here as well that this high constitutive activity may also be influenced by interactions with cellular lipids.
Lines 402-404: I recommend rephrasing the entire sentence about the recognition of LPI. It is unclear how recognition can be divided into sections. Perhaps the molecule could be described in terms of chemically distinct parts?
Lines 413-414: A reference to the supplementary table is absent.
Line 634: The phrase “fitted into the consensus map” could be clearer. Please specify which map was used.

Thanks!

Reviewer #2

(Remarks to the Author)
This paper presents the first high-resolution cryo-EM structures of the orphan G protein-coupled receptor GPR55 in complex with its putative endogenous agonist lysophosphatidylinositol (LPI) and synthetic agonist ML184, both bound to a G13 protein. The structures reveal novel insights into ligand recognition, receptor activation, and G protein coupling for GPR55. The study identifies important features such as the role of N-linked glycosylation, a potential lipid entry route, and a cholesterol molecule interacting with ML184. The thorough characterization of the G protein interface provides valuable information on selectivity.

The authors could enhance the study by comparing their active state GPR55 structure with an inactive state model generated by AlphaFold, which could provide insights into conformational changes during activation. They could also explore the identified ML184 binding pose in relation to structure-activity relationship (SAR) data available in the literature, which would contextualize their findings within existing knowledge. Furthermore, an explanation of ML184's agonistic activity based on its interactions with GPR55, compared to the structurally similar antagonist ML193, would provide valuable insights into the structural determinants of ligand efficacy.

Overall, this is a strong paper that significantly advances our understanding of GPR55 structure and function, opening new avenues for drug design and contributing to our knowledge of lipid-sensing GPCRs. The suggested additions would further increase its impact.

Version 1:

Reviewer comments:

Reviewer #1

(Remarks to the Author)
The authors have adequately addressed all my concerns and, where necessary, provided clear explanations of their perspectives. I recommend publishing the revised version as soon as possible.

Reviewer #2

(Remarks to the Author)
The authors have addressed all my comments. I have nothing to add.

Open Access This Peer Review File is licensed under a Creative Commons Attribution 4.0 International License, which permits use, sharing, adaptation, distribution and reproduction in any medium or format, as long as you give appropriate credit to the original author(s) and the source, provide a link to the Creative Commons license, and indicate if changes were made.
In cases where reviewers are anonymous, credit should be given to 'Anonymous Referee' and the source.
The images or other third party material in this Peer Review File are included in the article’s Creative Commons license, unless indicated otherwise in a credit line to the material. If material is not included in the article’s Creative Commons license and your intended use is not permitted by statutory regulation or exceeds the permitted use, you will need to obtain permission directly from the copyright holder.
To view a copy of this license, visit https://creativecommons.org/licenses/by/4.0/

REVIEWER COMMENTS

Reviewer #1 (Remarks to the Author):

In their study, Tobias Claff et al. investigated the structures of the orphan lipid receptor GPR55, an emerging drug target, in complex with a putative endogenous agonist, 1-palmitoyl-2-lysophosphatidylinositol (LPI), and a synthetic agonist, ML184. The authors expand their structural findings with data from G protein recruitment experiments, incorporating various point mutations and molecular dynamics simulations to validate the structures and further explore the interaction interfaces. Notably, the ligand density in the LPI-bound structure suggests a direct interaction between the ligand and an N-linked glycan. Furthermore, in both structures, the ligands face the lipid bilayer, with a cholesterol-like binder attached to the receptor that interacts with the ligands. The structures of GPR55 have not been published before, making these insights novel and certainly deserving of publication in Nature Communications.

The paper is well-written, the figures are clear, and the densities obtained by cryo-EM are of high quality, accurately reflecting the experimental data. I did not identify any seriously misleading elements in the manuscript; however, I strongly recommend addressing several points before publication.

Thank you very much for the overall very positive evaluation of our manuscript. The time you took for this comprehensive review is very much appreciated.

Major Points:

The contact between the modelled cholesterol and ligands is discussed repeatedly in the manuscript. However, cholesterol hemisuccinate tris (CHS-Tris) was present during solubilization and in all purification buffers, including the final size-exclusion buffer. It is plausible that a CHS molecule could bind instead of cholesterol, given these conditions. The structural difference between cholesterol and CHS lies in a distant region of the molecule that may not be fully resolved. Please clarify why cholesterol is present unambiguously or rephrase where necessary.

We now additionally have refined the complex with CHS instead of CLR. A superposition of the CHS (yellow) and CLR (green) binding poses is attached below showing virtually identical binding poses. It is true that no evidence for the hemisuccinate substitution can be found in

the cryo-EM map. Therefore, we have not replaced CLR by CHS in the deposited atomic model. However, we agree that CHS is the most likely option for binding in this pocket in our experimental conditions. Therefore, as suggested, we have explained the possibility of CHS in the main text and rephrased the following sentences:

"Remarkably, the ML184-structure revealed density that can be attributed to a cholesterol (CLR) molecule blocking the membrane opening within a cleft formed by helices IV and V (Fig. 2e, Fig. 3b and c, Supplementary Fig. S3 and S7). ***Admittedly, the CLR content of the employed insect cell membranes is significantly lower than that of mammalian cells[50] and the CLR dicarboxylic acid monoester, cholesteryl hemisuccinate (CHS), was employed during purification. Therefore, CHS may likely be bound to the receptor instead of CLR. However, the cryo-EM map does not provide additional evidence for the esterification (Supplementary Fig. S3) and consequently, CLR was modeled.****"*

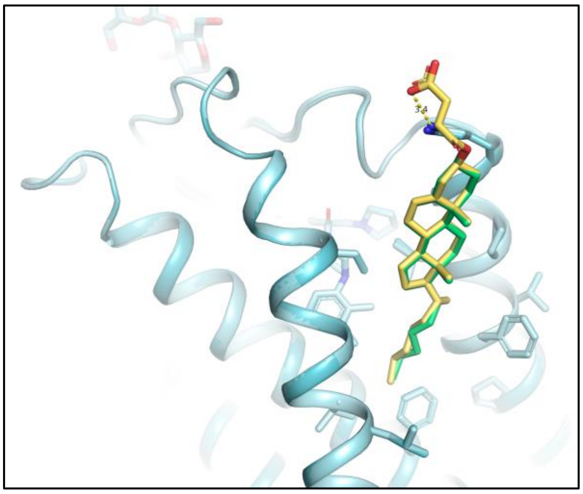

While G protein dissociation data is valuable, it should be interpreted with caution. Differences between mutants may be influenced by variations in expression levels. Especially, when glycosylation is affected (which might alter receptor folding and trafficking). This concern does not apply to EC50 values, but efficacy and basal activity values may have been affected. Was there a control for mutant expression/surface expression levels? If not, please, state it in the text and make it explicit that the data might have been affected. If yes, please, provide the data.

Thank you very much for bringing up this very important point. In this case, we have used wt GPR55 constructs without the addition of any tags for the functional characterization with the G protein dissociation assay. Therefore, efforts to investigate the surface expression of the GPR55 mutants were limited to antibodies recognizing the receptor sequence. The transfected amount of receptor DNA within the G protein dissociation assay is comparatively low (150 ng

DNA per 1,000,000 cells), and we expect relatively low receptor expression. Unfortunately, attempts to verify the receptor expression with GPR55-specific antibodies were not successful (e.g. via western blotting). Therefore, we relied on performing internal control experiments within the G protein dissociation assay, e.g., by employing the thromboxane A2 receptor and its high affinity ligand U-46619 that validate the successful surface expression of GPR55 and specific receptor activation with ML184 and LPI. However, it is true that we cannot ensure equal expression of all mutants, which might potentially slightly affect EC_{50}, efficacy, and basal activity values. It is fortunate that none of the mutations completely abolish receptor activity, and all mutations can be activated by at least one of the investigated ligands which ensures that all mutations are indeed expressed to some extent on the HEK cell surface. Additionally, the effect of some important mutations cannot be attributed to differential receptor expression, e.g., the R253A or G152W mutation which show completely opposite effects with both ligands. The afore-mentioned glycosylation mutation certainly is critical and receptor expression might indeed be affected, although GPR55 still contains another putative glycosylation site within its N-terminus. We have now carefully reviewed the manuscript and added statements to the possibility of unequal receptor expression and its possible effects at two sections:

1. In the results section where the first mutant (R253A) is discussed:

"*Notably, for G protein dissociation assays with wt GPR55 and its mutants, we employed untagged native receptor sequences which prevented the quantification of their surface expression in HEK cells. We cannot exclude the possibility that differential receptor expression may potentially affect the comparison of ligand potency and efficacy as well as basal receptor activation.*"

2. In the discussion where the first mutants are discussed:

"Admittedly, even single point mutations can affect the receptor's surface expression and differential receptor expression may potentially influence the comparison of ligand potency and efficacy or the basal receptor activation. However, none of the investigated GPR55 mutations was completely inactive and some mutations (e.g. R253$^{6.62}$A or G152$^{4.56}$W) even showed opposite effects on the potency of both ligands which cannot be explained solely by altered receptor expression."

Although not a major issue, there is a sense that with such a high starting number of micrographs and particles, the resolution of the structures could be pushed even further. It appears that the authors have not attempted to correct for motion per particle (e.g., Bayesian polishing in Relion or Reference-Based Motion Correction in recent CryoSPARC versions). Additionally, CTF refinement, per-particle defocus refinement, and splitting into AFIS groups>separately correcting for the beam tilt, etc. might improve the resolution. It is not uncommon to achieve a resolution improvement of 0.1-0.3 Å using some or all of these techniques.

Thank you very much for the additional processing suggestions! As an example, we have reprocessed the ML184 dataset using local & global CTF refinement, and also employed Reference-based Motion Correction on top. The downstream processing workflow was repeated as described in Supplementary Fig. S2 (Non-uniform refinement, and two subsequent local refinements). Below, we compiled a figure comparing the resolution plots of the NU-refinement and the final local refinement jobs. Indeed, as suggested, the final resolution of the maps after the NU-refinement and local refinement jobs improved by ~0.25 Å when Local and Global CTF refinement was combined with Reference-based Motion Correction, whereas the CTF refinement jobs alone only marginally improved the resolution.

To verify whether the nominally better effect also resulted in an improvement in map quality, we compared the original map and the map after CTF-refinement and Reference-based Motion Correction within selected areas of the protein (orthosteric pocket, CLR binding site, G protein site and ScFv16). In this instance, however, we feel that the final map quality is comparable between these jobs. Therefore, the new maps would not guide us to build a different or better model. We highly appreciate the suggestion to adapt our processing strategy. As we do not see any major impact on the quality of EM Maps, we would like to keep the maps as deposited in the PDB.

Further, we have repeatedly tested the proposed methods on different targets internally in the past in the hope of optimizing the map quality for modeling ligands, but our conclusion was that the benefit is usually not worth the additional computation time. We explain this finding by a very stable data acquisition and the use of the improved methods for motion correction and CTF-estimation in CryoSPARC, which already consider the anisotropic motion and deformation in the sample by tiling the micrographs into small patches (e.g. Patch Motion Correction, Patch CTF Estimation). This typically reduces the effect of additional correction methods such as Bayesian Polishing, local CTF refinement or Reference-based Motion Correction. Nevertheless, your valuable suggestion has convinced us to test these processes

continuously in the future - as an additional comparison and as an ongoing learning experience.

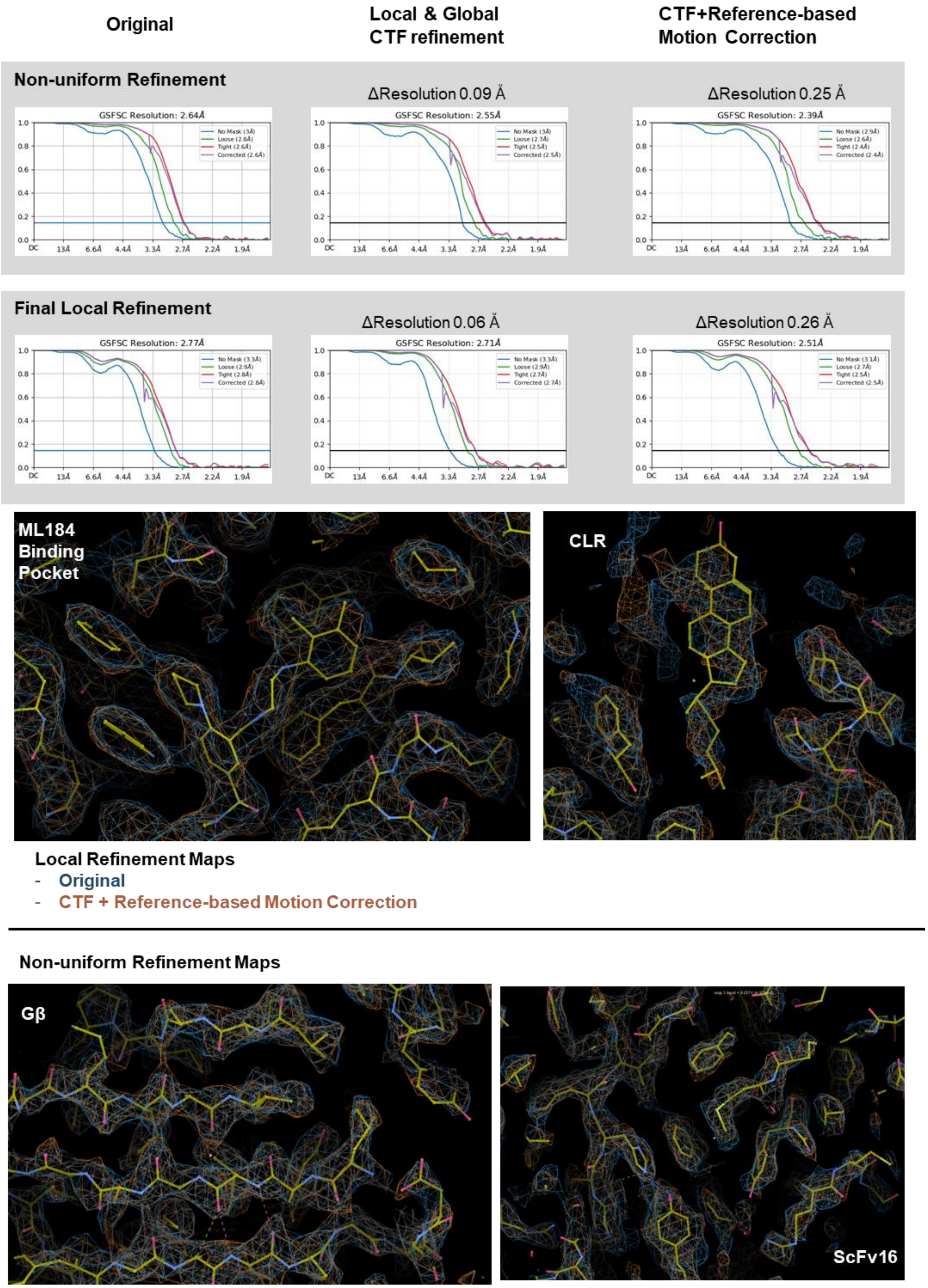

Minor points:

I suggest adding the tissue expression pattern of GPR55 to the introduction for context.

Information about the tissue expression of GPR55 has been added to the introduction:

"GPR55 is highly expressed in adrenal tissue and several brain areas but also in the gastrointestinal tract, liver and on immune cells[11,13,14]."

How similar are the two solved structures? What is the root mean square deviation (r.m.s.d.) between them?

This information has now been added to the section discussing the GPR55 activation and G protein interface:

"The two structures of GPR55 were determined in the G protein-bound active state and are highly similar with a RMSD of 1.91 Å considering all atoms and 1.42 Å within the G protein heterotrimer."

To be precise, I recommend replacing "endogenous ligand" (referring to LPI) with "putative endogenous ligand."

Thank you, in many cases we had already referred to LPI as the putative endogenous ligand but there were a few missing statements. We have now corrected these and added "*putative*" to the following sentences:

1. Abstract: "Notably, the **putative** endogenous ligand is anchored to the receptor […]"
2. Introduction: "Notably, our work delivers a structural understanding of the role of LPI as **putative** endogenous activator of GPR55 […]"
3. Results headline: "The binding mode of the **putative** endogenous ligand elucidates crucial roles […]"

In the section discussing GPR55 activation, only the higher-resolution structure is considered. There is no mention of the activation microswitches comparison between the two solved structures. Are they the same? This information would be interesting to include.

The conformation of the common activation microswitches is virtually identical between both structures and we have now added the information to the manuscript. However, to not

compare even more structures in Fig. S8, we propose to compare the higher resolution structure still alone with the other GPCR structures. The section now reads as follows:

"The two structures of GPR55 were determined in the G protein-bound active state and are highly similar with an RMSD of 1.91 Å considering all atoms and 1.42 Å within the G protein heterotrimer. The common activation microswitches of GPR55 were found to contain features of an activated conformation[51] (Supplementary Fig. S8). Their arrangement is virtually identical between both structures, but for clarity, only the higher resolution structure in complex with ML184 is shown. Notably, the classical activation motifs described for class A GPCRs are not fully conserved in GPR55[52]."

I recommend revisiting the modelled water molecules to take a more conservative approach, considering densities on the focused map and the local resolution of the region. At least two of them, HOH5 and HOH6 (as shown in the attached figures), appear to have very weak densities compared to the others and the neighbouring side chains.

Thank you very much for revisiting the modelled water molecules! We agree that the map quality for HOH6 is very weak. Additionally, the area of the structure is surface exposed, and the coordination is not optimal. Therefore, we have initiated the removal of HOH6 from the PDB model.

The cryo-EM map for HOH5 is also weaker than the surrounding water molecules indicating some residual flexibility. Nevertheless, we think that its position can be well explained by its coordination with R119, helix VI, and VII in the available structural space. We propose to clarify the weaker density and its implications in the figure caption:

*"The orange mesh in panel **e** shows the cryo-EM composite map (EMDB-51284) of five water molecules. The map quality of one water molecule deviates significantly from four well resolved water molecules. The modelling of this water molecule was guided by its coordination with R119$^{3.50}$, helix VI, and helix VII."*

Additionally, to be completely transparent, we have added a comment to the water molecule that promotes the interaction of LPI with the GlcNAc of N-linked glycosylation:

"This region is located at the receptor surface showing lower resolution and possibly higher flexibility. Consequently, the structural water molecule could be transient with a high bulk water exchange rate."

While not essential, it would be beneficial to include a comparison with the positions of

canonical activation switches (such as in the β2 adrenergic receptor) if claiming that the activation pathway differs from the canonical one.

Thank you very much for this suggestion! We have now adopted Supplementary Fig. S8 to show the inactive and active states of the β_2 adrenergic receptor (PDB IDs 2RH1 and 3SN6). We also think this comparison is more insightful than the comparison with the inactive state structures of the S1PR and TBXA2R. Therefore, the comparison with these receptors was removed in the final Supplementary Figure S8. These adjustments helped us to notice that the activation microswitches between the β_2 adrenergic receptor and GPR55 are quite similar while the helix outward movements are more distinct. Taking these new observations into account, we have revisited the whole paragraph and attenuated the statement about the difference from the canonical activation mechanism. Additionally, based on a comment of Reviewer 2, we have included a comparison with an inactive state AlphaFold2 model.

"These microswitches are more conserved between GPR55 and GPR35 (Supplementary Fig. S8f) and the recently determined active state structure of GPR35 in complex with G_{13} was therefore used for comparison[41]. Additionally, we compared GPR55 to an inactive state AlphaFold2 model[53,54] of GPR55 (downloaded from GPCRdb[55]), and to inactive[56] and active[57] state structures of the prototypical β_2 adrenergic receptor (β_2AR) although the receptors do not share identical motifs (Supplementary Fig. S8f). Notably, both GPR55 and GPR35 exhibit virtually identical activated microswitch conformations, consistent with the active state of the β_2AR. However, GPR55 and GPR35 contain the transmission switch residue $F^{6.48}$ instead of $W^{6.48}$ and only show small helix VI outward movements (4-5 Å) when compared to the large 14 Å switch of the β_2AR (Supplementary Fig. S8). Within this comparison, the intracellular end of helix I shifts outward, and helix VII moves into the G protein binding site as similarly observed for the β_2AR. In contrast, helix V of GPR55 and GPR35 is practically stationary during activation, whereas helix V of the β_2AR shows an additional outward movement. The inactive state AlphaFold2 model of GPR55 is moderately similar to the active GPR55 cryo-EM structure (RMSD 3.3 Å) and aligns well with the overall conformation of the inactive β_2AR (Supplementary Fig. S8), supporting the predicted conformational changes. These results indicate that the underlying activation mechanism of GPR55 (and GPR35[41]) seems to have features related to classical GPCR activation,[51,52] but also aspects that enable $G\alpha_{13}$ binding without large helix V and VI outward movements. The determination of inactive state structures of the same receptor will be instrumental to further explore the mechanism of GPR55 activation."

Another example of a lipid receptor with a suggested opening between helices IV and V is the CysLTR1 receptor. As far as I recall, there were even molecular dynamics simulations of the lateral ligand entrance (Luginina et al. 2019).

Thank you very much for pointing us towards the CysLTR1 publication where a similar membrane opening between helices IV and V was observed. Here, the authors also hypothesize a lateral ligand entry for a synthetic ligand and a collapse of the membrane opening without ligand was observed in molecular dynamics simulations. Although the overall research question in Luginia et al. was somewhat different and more ligand focused, the published methodology is similar to our molecular dynamics simulations, and with a similar hypothesis in regards to ligand entry. We therefore have added a citation to Luginina et al. 2019 at two respective parts in the manuscript:

1. In the discussion where we hypothesize lateral ligand entry:

Recently, the same observation was also made for oleic acid at GPR3[66–68] ***and for the synthetic cysteinyl leukotriene receptor antagonist zafirlukast***[**70**]*. For both receptors, a similar access route for ligands to the binding pocket from the membrane was hypothesized*[66,**70**]*."*

2. In the discussion where the importance of CLR for stabilizing the extracellular portion around the membrane opening is discussed:

"Relatedly, in MD simulations of the cysteinyl leukotriene receptor 1, it was previously demonstrated that the presence of an orthosteric ligand is essential to prevent the collapse of a similar membrane opening between helices IV and V[70]*."*

Figures:

Main Figure 1 (a and e): The transparent density at a different contour level (likely due to the micelle visualization) is somewhat confusing, particularly the top blobs that originally belonged to the protein. I encourage the authors to explore options in Chimera to mitigate this effect.

The transparent cryo-EM maps at lower contour levels represent the original density to visualize the micelle but also to show the lower resolution parts of the GPCR, G protein and ScFv16. This visualization is entirely without map modifications, e.g., removal of dust or erasing volumes. Although we are aware of the capabilities of Chimera to modify cryo-EM maps for visualization purposes, we here prefer to show the original map without modifications. In particular, the top blobs that protrude from the micelle belong to low resolution

density of helix I and to the cryo-EM map of receptor glycosylation, respectively, which hopefully should become clearer when comparing the maps in panels a and e to the respective models in panels c and f.

However, we have now specified the two different contour levels in the figure caption:

"**Fig. 1. Architecture of the GPR55-G$_{13}$ signaling complex with lipid and synthetic agonists. a** Cryo-EM map (consensus**, EMDB-51285**) of the GPR55-G$\alpha_{13}\beta_1\gamma_2$-ScFv16-LPI complex **at two different contour levels**. The enlarged cryo-EM map for LPI (yellow sticks) is shown in blue mesh. **b** Full model corresponding to the signaling complex of **a** (shown as cartoon representation). **c** Chemical structure of LPI. **d** Overview of ligand binding pocket position of LPI. **e** Cryo-EM map (consensus**, EMDB-51281**) of the GPR55-G$\alpha_{13}\beta_1\gamma_2$-ScFv16-ML184 complex **at two different contour levels**."

Main Figure 2 (a): Please add a label for residue 171, as described in the text.

The missing label for N171^{ECL2} has now been added. Thank you!

Main Figure 5 (e): Please specify which map is depicted.

The map depicts the final composite map. The information has now been added to the figure caption.

*"The orange mesh in panel **e** shows the cryo-EM composite map (EMDB-51284) of five water molecules."*

Line by line:

Line 10: It appears that "activation of lipid GPCRs" or something similar is missing. The sentence does not seem complete.

We agree that this statement is not true for all GPCRS and there added "lipid GPCRs" for clarification. The sentence now reads as follows:

"The endogenous activation of lipid GPCRs can be solely mediated by membrane components and different lipids have been proposed as endogenous activators of GPR55, such as cannabinoids and lysophosphatidylinositols."

Line 16: I suggest removing “(endogenous)” for clarity.

“(endogenous)” has been removed.

Line 39: A reference to the reported selective G13 coupling of GPR55 is missing.

Two references (Inoue et al. 2019 and Ryberg et al. 2007) have been added to corroborate the statement.

Line 357: While you mention this in the discussion, it would be helpful to note here as well that this high constitutive activity may also be influenced by interactions with cellular lipids.

Thank you for the suggestion. We have added a statement accordingly:

“Herein, we observed robust G protein activation (concluded from a $BRET^2$ ratio decrease) when GPR55 was co-expressed with $G\alpha_{13}$ (Fig. 5b), even without the addition of any ligand. (Fig. 2f, Supplementary Table S3). This observation is indicative of constitutive activity; however, it could also be explained by GPR55 interaction with ubiquitously present cellular lipids.”

Lines 402-404: I recommend rephrasing the entire sentence about the recognition of LPI. It is unclear how recognition can be divided into sections. Perhaps the molecule could be described in terms of chemically distinct parts?

We agree that this is somewhat confusing and adopted the reviewer’s suggestion to highlight the chemically distinction of the ligand itself:

“The structure of LPI can be divided into three chemically distinct sections that shape its recognition at GPR55: the hydrophilic phosphoinositol head group, the glycerol moiety, and the lipophilic palmitoyl tail.”

Lines 413-414: A reference to the supplementary table is absent.

A reference to Supplementary Table S4 has been added.

Line 634: The phrase "fitted into the consensus map" could be clearer. Please specify which map was used.

We have specified the map used for fitting of the initial model and for the subsequent refinements and added the specific EMDB-codes for clarity:

"The AlphaFold2 model[75] of GPR55 and the $G\alpha_{13}\beta_1\gamma_2$-ScFv16 complex [from Protein Data Bank (PDB) ID 7YDH[76]] were fitted into the ***cryo-EM consensus maps (EMDB-51285 and EMDB-51281)*** *as an initial model using ChimeraX[73]. Iterative cycles of model building via Coot[77] and real-space refinements using Phenix[78]* ***using the final composite maps (EMDB-51288 and EMDB-51284)*** *were performed to improve the model."*

Thanks!

We thank you for your time to conduct this comprehensive review. Your comments were very effective in improving the clarity of the manuscript.

Reviewer #2 (Remarks to the Author):

This paper presents the first high-resolution cryo-EM structures of the orphan G protein-coupled receptor GPR55 in complex with its putative endogenous agonist lysophosphatidylinositol (LPI) and synthetic agonist ML184, both bound to a G13 protein. The structures reveal novel insights into ligand recognition, receptor activation, and G protein coupling for GPR55. The study identifies important features such as the role of N-linked glycosylation, a potential lipid entry route, and a cholesterol molecule interacting with ML184. The thorough characterization of the G protein interface provides valuable information on selectivity.

The authors could enhance the study by comparing their active state GPR55 structure with an inactive state model generated by AlphaFold, which could provide insights into conformational changes during activation.

Thank you very much for your comment. Based on your suggestion, we have used the AF-2 model of inactive GPR55 (downloaded from GPCRdb) and compared it to our GPR55 structure and the inactive state structure of the β_2AR (PDB ID 2RH1). Based on a comment of Reviewer 1, we have already modified Supplementary Fig. S8 to include inactive and active state β_2AR structures (see response above). This figure now also includes the superimposition

of the inactive state AF-2 model (rectangle boxes). We found that the overall architecture of the inactive state model aligns well with the inactive state of the β_2AR. Therefore, the conclusions regarding conformational changes during activation are consistent with the β_2AR comparison that we have added based on the comments of Reviewer 1. In addition, have now also added the reference to the AF-2 model and the respective RMSD between our structure and the inactive state model. For your convenience, we will copy the complete new section below:

"The two structures of GPR55 were determined in the G protein-bound active state and are highly similar with an RMSD of 1.91 Å considering all atoms and 1.42 Å within the G protein heterotrimer. The common activation microswitches of GPR55 were found to contain features of an activated conformation[51] (Supplementary Fig. S8). Their arrangement is virtually identical between both structures, but for clarity, only the higher resolution structure in complex with ML184 is shown. Notably, the classical activation motifs described for class A GPCRs are not fully conserved in GPR55[52]. For instance, instead of the CWxP, DRY, NPXXY, and PIF motifs, GPR55 carries SFxP, DRF, DVxxY, and PVF motifs. These microswitches are more conserved between GPR55 and GPR35 (Supplementary Fig. S8f) and the recently determined active state structure of GPR35 in complex with G_{13} was therefore used for comparison[41]. ***Additionally, we compared GPR55 to an inactive state AlphaFold2 model[53,54] of GPR55 (downloaded from GPCRdb[55]),*** *and to inactive[56] and active[57] state structures of the prototypical β_2 adrenergic receptor (β_2AR) although the receptors do not share identical motifs (Supplementary Fig. S8f). Notably, both GPR55 and GPR35 exhibit virtually identical activated microswitch conformations, consistent with the active state of the β_2AR. However, GPR55 and GPR35 contain the transmission switch residue $F^{6.48}$ instead of $W^{6.48}$ and only show small helix VI outward movements (4-5 Å) when compared to the large 14 Å switch of the β_2AR (Supplementary Fig. S8). Within this comparison, the intracellular end of helix I shifts outward, and helix VII moves into the G protein binding site as similarly observed for the β_2AR. In contrast, helix V of GPR55 and GPR35 is practically stationary during activation, whereas helix V of the β_2AR shows an additional outward movement.* ***The inactive state AlphaFold2 model of GPR55 is moderately similar to the active GPR55 cryo-EM structure (RMSD 3.3 Å) and aligns well with the overall conformation of the inactive β_2AR (Supplementary Fig. S8), supporting the predicted conformational changes.*** *These results indicate that the underlying activation mechanism of GPR55 (and GPR35[41]) seems to have features related to classical GPCR activation,[51,52] but also aspects that enable $G\alpha_{13}$ binding without large helix V and VI outward movements. The determination of inactive state structures of the same receptor will be instrumental to further explore the mechanism of GPR55 activation."*

They could also explore the identified ML184 binding pose in relation to structure-activity relationship (SAR) data available in the literature, which would contextualize their findings within existing knowledge.

Thank you very much for this suggestion. We could find one SAR study investigating seven compounds related to ML184 in Brown, A.J. et al. 2011 (https://doi.org/10.1124/jpet.110.172650) and hope that this is the suggested study you are referring to. Unfortunately, the authors did not investigate ML184 or compared their compounds to the agonist that we have used. The fact that this SAR investigation is not stringent, meaning that multiple modifications were introduced between different compounds (e.g. substitution pattern of the aromatic moiety in residue Y), additionally hampers a detailed analysis. Nevertheless, we added two references to the main text where they are applicable as follows:

- *"The pyrrolidine moiety of ML184 addresses a lipophilic sub-pocket formed by W177$^{5.34}$, F182$^{5.39}$, L185$^{5.42}$, and F246$^{6.55}$ (Fig. 2e). **Notably, a previous structure-activity relationship study investigating compounds related to ML184 found that larger aromatic residues seem to be tolerated in this position[36].**"*

- *The terminal dimethylphenyl ring is stabilized by aromatic stacking interactions with Y106$^{3.37}$ and Y157$^{4.61}$ (Fig. 2e) and points towards the same membrane opening between helices IV and V as observed for the lipid tail in the LPI-bound structure (Fig. 1g, 2c and 3). **Small and flexible o,p-disubstituted ML184-derivatives were demonstrated to be tolerated at this position whereas larger substituents or bicyclic systems reduced ligand potency[36].***

Furthermore, an explanation of ML184's agonistic activity based on its interactions with GPR55, compared to the structurally similar antagonist ML193, would provide valuable insights into the structural determinants of ligand efficacy.

Thank you for bringing ML193 to our attention. Both compounds are certainly somewhat similar, e.g., both contain a phenyl-sulfonamide moiety. We will provide the structure of both in an aligned fashion below. Additionally, we generated a 3D structure of ML193 and attempted to align both binding poses (ML193 and ML184) by aligning the phenyl-sulfonamide moieties. Unfortunately, the 3D structure of ML193 largely deviates from the orientation of ML184 and it is challenging to predict the binding pose of ML193 or even compare it with the binding pose

of ML184. Overall, we feel that we are unable to draw conclusions that might explain the antagonistic efficacy of ML193 in comparison with the agonistic efficacy of ML184. Therefore, we would not include this comparison in the manuscript.

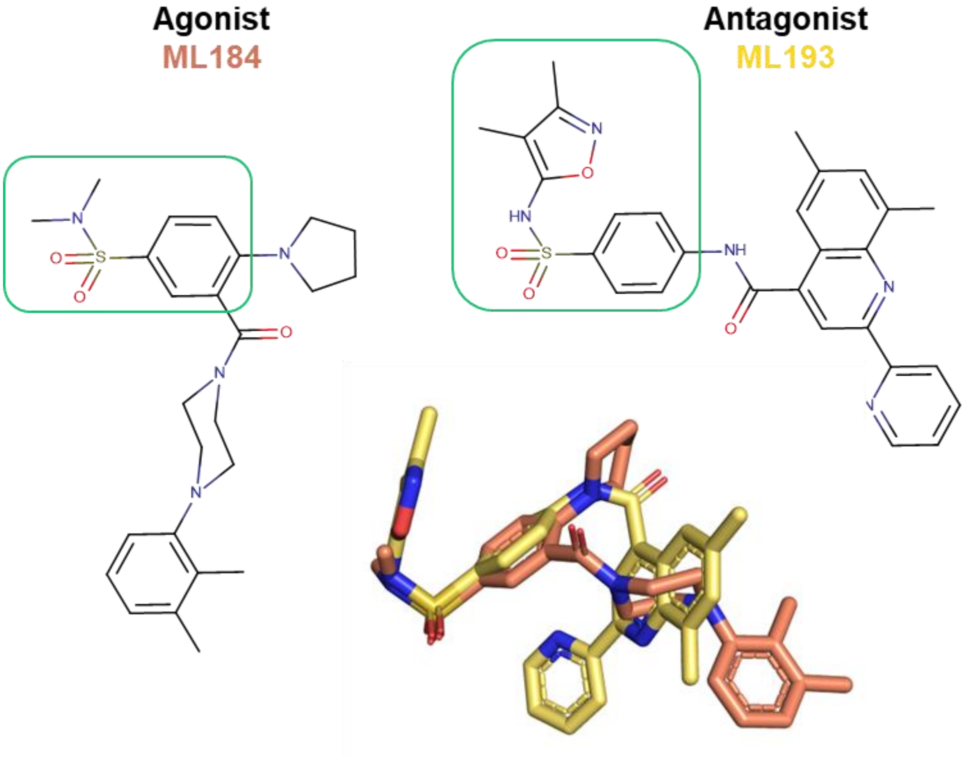

Overall, this is a strong paper that significantly advances our understanding of GPR55 structure and function, opening new avenues for drug design and contributing to our knowledge of lipid-sensing GPCRs. The suggested additions would further increase its impact.

Thank you very much for the overall positive evaluation of our work! We appreciate your time in reviewing the manuscript.

Additional minor modifications:

- Line 467: “Therefore” was changed to “In conclusion”

- Line 469: “Overall” was removed

- Line 692f: The number of cells used for HEK transfection was not accurate and has been corrected to 1,000,000 cells per well instead of 1,500,000 cells.

- A typo in Supplementary Table S3 was corrected. The pEC_{50} of ML184 at wt GPR55 was determined to be 7.35 instead of 7.36. The respective P values were corrected as well without affecting the overall conclusions.

- Supplementary Fig. S4 was corrected: Plots for GPR55-wt and for the TBXA2R were removed as they are already shown in Main Text Fig. 5. Instead, the so far missing plots for the four G13 mutants are now included (L374I, Q373D, H368Q, and R331D). Additionally, we have harmonized the y-axis scale of all plots for better comparability:

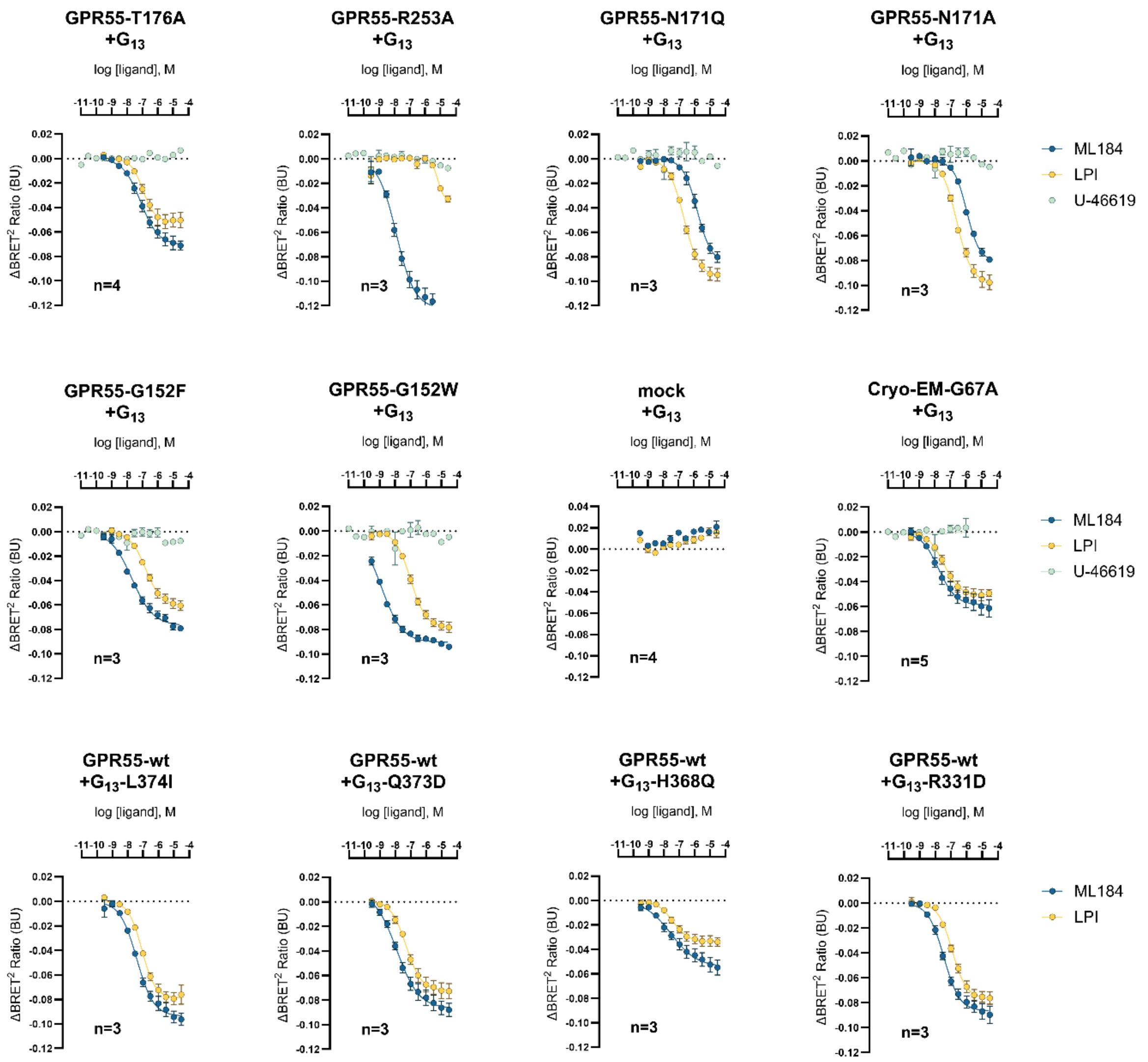

Supplementary Fig. S4. Dose-response curves of G protein dissociation assays. Data points represent means ± SEM from 3-7 independent experiments as indicated in Supplementary Table S3. Data of the negative control ligand U-46619 at GPR55 mutants represent means ± SEM from only two independent experiments.

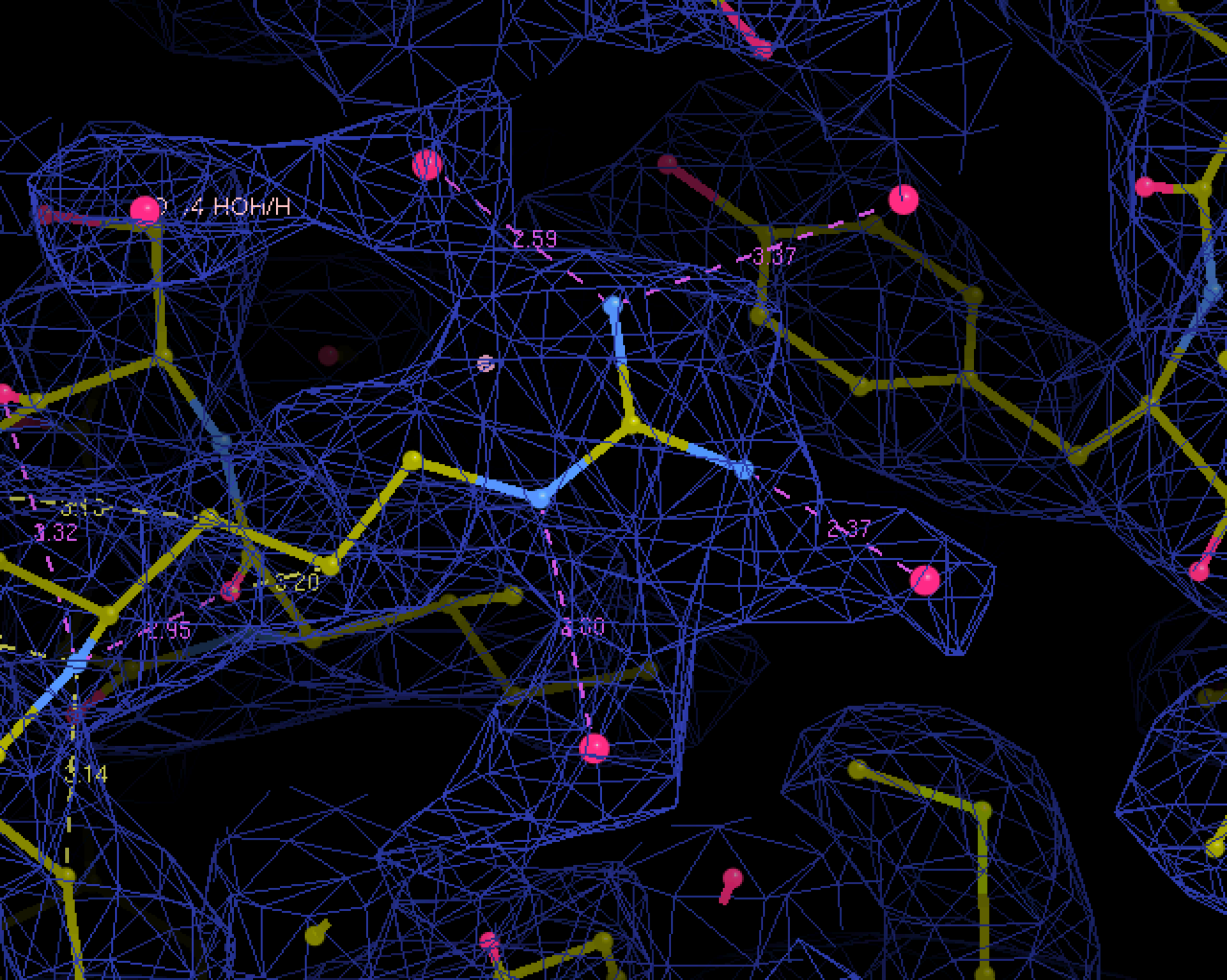
2.59
3.37
2.37
3.32
2.95
3.14

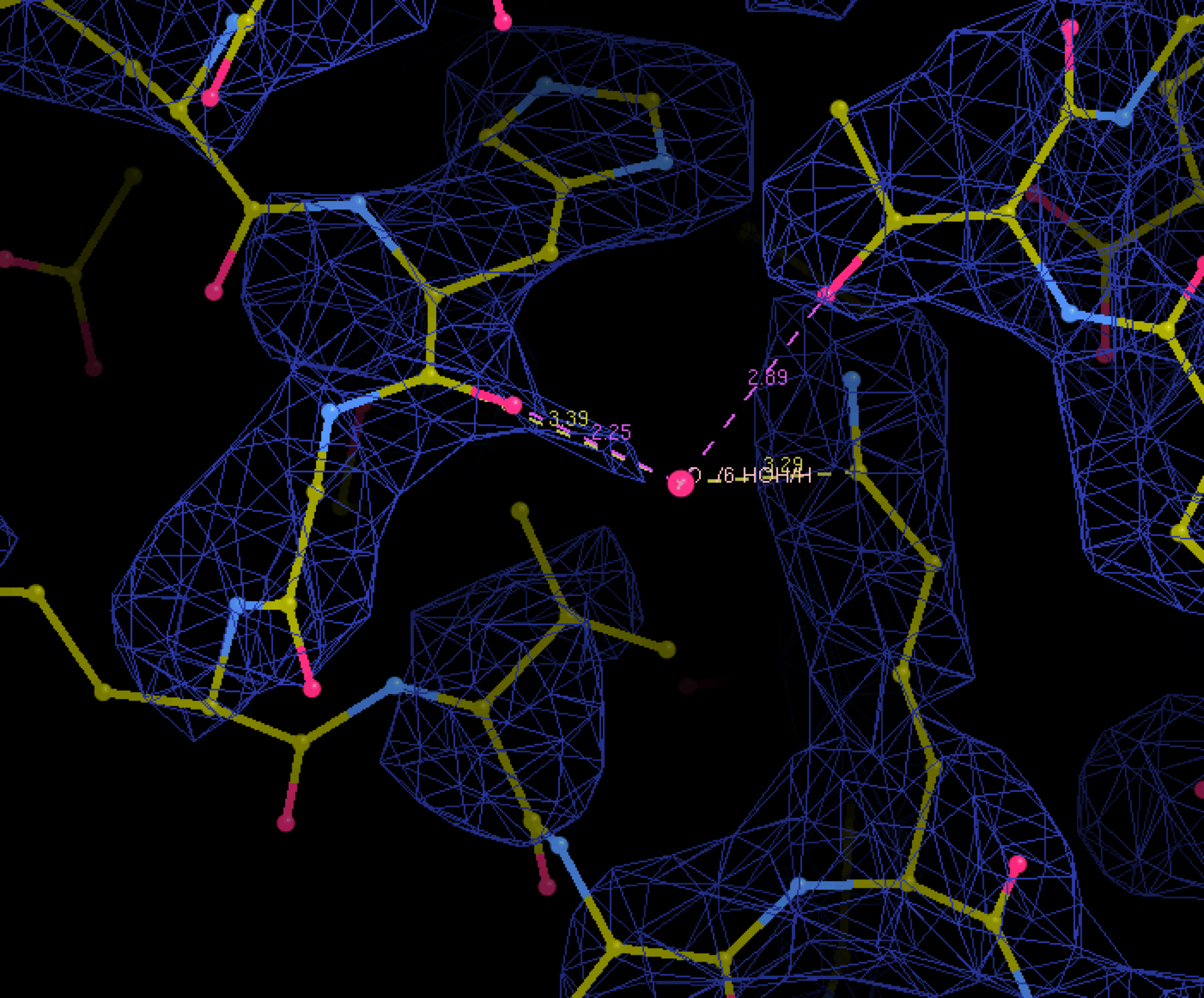
2.89
3.39
2.25
3.29
O /6 HOH/H
